# Maternal group B *Streptococcus* decreases infant length and alters the early-life microbiome: a prospective cohort study

**DOI:** 10.1080/07853890.2024.2442070

**Published:** 2024-12-18

**Authors:** Shanshan Li, Qijun Liang, Wei Qing, Zhencheng Fang, Chunlei Yuan, Shilei Pan, Hairui Xie, Xiaocong Li, Muxuan Chen, Yan He, Hongwei Zhou, Qian Wang

**Affiliations:** a Department of Laboratory Medicine, Microbiome Medicine Center, Zhujiang Hospital, Southern Medical University, Guangzhou, Guangdong, China; b Department of Obstetrics and Gynecology, Boai Hospital of Zhongshan, Zhongshan, Guangdong, China; c Department of Laboratory Medicine, Boai Hospital of Zhongshan, Zhongshan, Guangdong, China; d Department of Obstetrics and Gynecology, Zhujiang Hospital, Southern Medical University, Guangzhou, China; e Department of Paediatrics Center, Zhujiang Hospital, Southern Medical University, Guangzhou, Guangdong, China; f Shenzhen Stomatology Hospital (Pingshan), Southern Medical University, Guangdong, China

**Keywords:** Group B *Streptococcus*, vaginal microbiota, infant growth, length-for-age *z-*score, gut microbiota

## Abstract

**Background:**

Maternal colonization with Group B Streptococcus (GBS) disrupts the vaginal microbiota, potentially affecting infant microbiota assembly and growth. While the gut microbiota’s importance in infant growth is recognized, the specific effects of maternal GBS on growth remain unclear. This study aimed to explore the effects of maternal vaginal GBS during pregnancy on early infant growth, microbiome, and metabolomics.

**Methods:**

We recruited and classified 453 pregnant women from southern China into GBS or healthy groups based on GBS vaginal colonization. Their infants were categorized as GBS-exposed or GBS-unexposed groups. We comprehensively analyzed infant growth, gut microbiota, and metabolites during early life, along with maternal vaginal microbiota during pregnancy, using 16S rDNA sequencing and targeted metabolomics.

**Results:**

GBS-exposed infants exhibited lower length-for-age z-scores (LAZ) than GBS-unexposed infants, especially at 2 months. Altered gut microbiota and metabolites in GBS-exposed infants correlated with growth, mediating the impact of maternal GBS on infant LAZ. Changes in the vaginal microbiota of the GBS group during the third trimester correlated with infant LAZ. Additionally, differences in neonatal gut microbiota, metabolites, and vaginal microbiota during pregnancy were identified between infants with overall LAZ<-1 within 8 months after birth and their counterparts, enhancing the discriminatory power of fundamental data for predicting the occurrence of LAZ<-1 during the first 8 months of life.

**Conclusions:**

GBS exposure is associated with decreased infant length growth, with altered microbiota and metabolites potentially mediating the effects of maternal GBS on offspring length growth, offering potential targets for predicting and addressing growth impairment.

## Introduction

Ending stunting and wasting in children younger than 5 years is a key objective outlined in Sustainable Development Goal 2.2. To improve perinatal outcomes, intrapartum antibiotic prophylaxis (IAP) is offered to women identified as carriers of GBS and those with additional risk factors for intrapartum GBS colonization [1,2]. As a result, there was an approximately 75% reduction in neonatal early-onset GBS disease [3–6], with 19.3 million infants (98% of those exposed to maternal GBS) avoiding invasive GBS disease in 2020 [7]. Although the correlation between GBS-specific IAP and infant weight is controversial [8,9], there is currently no existing evidence demonstrating whether maternal GBS impacts offspring growth, despite GBS being present in the healthy placenta and potentially playing a role in the establishment and priming of immune competency [10,11].

Numerous studies have highlighted the influence of the gut microbiome on infant growth. Gut transitional *Bifidobacterium longum* and associated metabolites have been implicated in early growth, and their effects are achieved through enzymes that utilize breast milk and solid food substrates [12]. The immature gut microbiota of undernourished infants transmits impaired growth in mice, encompassing issues such as weight gain and bone morphology. Interestingly, the introduction of *Ruminococcus gnavus* and *Clostridium symbiosum* has been shown to ameliorate these growth abnormalities [13]. Furthermore, targeted microbiota-directed dietary interventions have proven advantageous for infant growth, including weight-for-length *z*-score (WLZ), weight-for-age *z*-score (WAZ) and proteins involved bone growth and neurodevelopment in children aged 12–18 months [14]. In a similar vein, indole-3-lactic acid derived from *Bifidobacterium infantis* has been shown to upregulate immunoregulatory galectin-1 in Th2 and Th17 cells during polarization, establishing a functional link between microbes and immunoregulation [15]. Therefore, understanding the infant gut microbiota and metabolites may aid in understanding the effects of maternal GBS on offspring growth [16]. These understanding are pivotal in unravelling the complex interplay between microbial dynamics and early-life growth outcomes.

The vaginal microbiota serves as the primary maternal microbial reservoir colonized by newborns during vaginal birth [17–19] and is correlated with infant growth. Intriguingly, in a mouse model, the lasting effect of transferring human vaginal microbiota (VMT) during birth on the weight of offspring from weaning to adulthood has unveiled. Specifically, body weight is significantly greater in males exposed to primary community state type (CST) IV than in those exposed to CST I [20]. Similarly, a randomized clinical trial indicated that VMT reduces the risk of overweight/obesity in infants born *via* caesarean section at 6 months of age [21]. Our previous study additionally illustrated that VMT not only enhances neurodevelopment but also accelerates the maturation of the gut microbiota, regulates the levels of specific faecal metabolites and metabolic functions in infants born through caesarean section [22]. Importantly, alterations in the vaginal microbiome have been reported in healthy pregnant women with GBS colonization in the third trimester [23]. However, the connection between modified vaginal microbiota and offspring growth has not been determined.

In this prospective cohort study conducted at two cities in southern China, we aimed to evaluate the previously undescribed impact of maternal GBS colonization in the third trimester on infant growth during the first 8 months of life. We collected faecal samples from offspring to analyze the gut microbiome and metabolome of infants exposed to GBS. Our investigation sought to provide preliminary insights into the role of these factors in influencing infant growth attributed to maternal GBS. Furthermore, we examined the association between the vaginal microbiota in the third trimester and infant growth by analyzing collected vaginal swabs and corresponding infant anthropometric data.

## Methods

### Ethics approval

This study adhered to the principles of the Declaration of Helsinki and received ethical approval from the Ethical Committee of Zhujiang Hospital of Southern Medical University (No.2022-KY-015-01), and Zhongshan Women and Children’s Hospital (No.2022-KY-004-02). The trial has been registered in the Chinese Clinical Trial Registry (chictr.org.cn, ChiCTR2200057279) and ClinicalTirals.gov PRS (clinicaltrials.gov, NCT05738473). All pregnant women participating in this study provided written informed consent.

### Study design and participant enrolment

This dual-centre prospective cohort study was carried out in Guangzhou and Zhongshan, southern China. Women in their third trimester were recruited from Zhujiang Hospital of Southern Medical University and Zhongshan Women and Children’s Hospital between February and December 2022. The research protocol received approval from the Ethical Committee of both hospitals.

Inclusion criteria:

1. Age 18–40 years;
2. 35 to 41 weeks of gestation;
3. Singleton pregnancy;
4. Planned natural labour and postpartum care at the study hospital;
5. Pregnant women who volunteered to participate in the study.

Exclusion criteria:

1. The serious infection and clinical disease;
2. Antibiotic administration within 2 weeks before enrolment;
3. Long-term medication for chronic diseases;
4. Vaginal douching or topical medication was administered within 24 hours before obtaining vaginal samples.
5. Any condition that the investigator considers inappropriate to participate.

Offspring were enrolled after birth. All mother-infant pairs underwent post-birth assessments, excluding infants: 1. Prematurely born or by caesarean section; 2. A birthweight of 4000 g ≤ or <2500 g or a diagnosis of severe disease; 3. Antibiotics were administered before researchers obtained qualified faeces.

### Sample collection and follow-up

Baseline information was recorded at the time of enrolment. Trained healthcare professionals collected vaginal secretion samples using aseptic procedures with two sterile swabs during enrolment. One swab was placed into a cryovial tube and stored at −80 °C until analysis, while another swab was inserted into a Microbiological Transport Swab (Cat: YP/S0051, IMPROVE, Guangzhou, China) for GBS culture.

Eligible mother-infant pairs were followed up for 8 months at 0, 1, 3, 6 and 8 months after birth. Demographic, clinical, feeding and anthropometric data were collected at each visit.

Approximately 1 g of infant faeces were collected at 2–3 days, and 2 months of age, the samples were subsequently transported to research centres with pre-frozen ice bags. Subsequently, the researchers divided the specimens into three tubes and stored them in a −80 °C freezer until analysis.

### Anthropometric measurements and calculations

Infant length, weight and head circumference were measured by trained child healthcare professionals at the two study hospitals. WHO Child Growth Standards (Anthro v3.2.2) were utilized to calculate the LAZ, WAZ, body mass index-for-age *z*-score (BMI *z*-score), WLZ and head circumference-for-age z-score (HCAZ). All eligible data were analyzed using R version 4.1.1.

### DNA isolation

DNA extraction from vaginal swabs and faeces was performed using QIAamp Micro Kit and QIAamp Fast DNA Stool Mini Kit (Cat: 56304/51604; Qiagen, Germany), respectively, following the manufacturer’s instructions. DNA concentrations were assessed using NanoDrop 2000 (ThermoFisher Scientific, USA).

### GBS testing

Assessment of GBS colonization was performed *via* both culture and Q-PCR methods.

The vaginal samples were cultured in selective enrichment Todd-Hewitt broth (Cat: LA1860; Solarbio, Beijing, China), GBS chromogenic plates (Autobio, Zhengzhou, China) and blood agar plates (Cat: LS2209; Detgerm, Guangzhou, China) in accordance with the guidelines of the American College of Obstetricians and Gynecologists committee [2]. The suspected GBS strains were identified using a MALDI-TOF MS system (Autof ms1000, Zhengzhou, China).

Q-PCR was conducted using AceQ qPCR SYBR Green Master Mix (Cat: Q111, Vazyme, Nanjing, China) on a Roche 480 instrument. The primers *cfb*-F 5′-GGGAACAGATTATGAAAAACCG-3′ and *cfb*-R 5′-AAGGCTTCTACACGACTACCAA-3′ were utilized to amplify the *cfb* gene of GBS in accordance with CDC recommendations.

### ELISA analysis

The concentrations of calprotectin and secretory immunoglobulin A (sIgA) (*n* = 361) were determined using enzyme-linked immunosorbent assays (ELISA, Cat: MM-0974H1/MM-2100H1; MEIMIAN, Jiangsu, China) following the manufacturer’s instructions.

### 16S rRNA gene sequence and analysis

The 16S rRNA gene was sequenced in faecal and vaginal samples using an Illumina NovaSeq sequencer (2 × 250 bp paired-end).

The detailed microbiome analysis methods can be found in Method S1 in the Supplementary Materials.

### Targeted metabolomic analysis

Targeted faecal metabolomics analysis of the faeces (*n* = 136) was conducted using a UPLC–MS/MS system from Metabo-Profile (Shanghai, China). Additional details can be found in the Supplementary Methods: Method S2.

### Statistical analysis

R (version 4.1.1) was used for statistical analysis. The demographic and clinical characteristics of the participants were compared using the descrTable function of the compareGroups package (version 4.5.1). For the infant growth comparisons, ANOVA, Wilcoxon and *t* test were used based on the data characteristics. Adjustment for infant growth analysis was performed with the aov function of the stats package (version 4.2.2). Spearman correlation analysis was conducted using the corr.test function in the psych package (version 2.2.9), and BH adjustment was applied for multiple tests. Mediation analysis was conducted using the mediation package (version 4.5.0). LASSO was conducted using glmne (version 4.1-4). The survival package (version 3.4-0) was used to assess the Cox model and test the proportional hazard assumption. Prediction models and model discriminations (C-index) were constructed using the glmnet and survival packages. ROC curves were generated using pROC (version 1.18.0). For data visualization, ggplot2 (version 3.3.5) and pheatmap (version 1.0.12) were utilized in RStudio (version 1.4.1106) under R.

## Results

### Participants and characteristics

From February to December 2022, we enrolled 453 women in their third trimester who underwent GBS testing. Initially, in conjunction with prior test results, we confirmed continuous GBS colonization in the maternal vagina using culture and quantitative real-time PCR (Q-PCR) methods. Subsequently, all women were classified into the GBS group or the healthy group. After delivery, infants in the GBS group were categorized as GBS-exposed due to maternal GBS exposure, while infants in the healthy group were classified as GBS-unexposed.

Ultimately, 1441 follow-up data from 387 infants aged 0–8 months were eligible for growth analysis after excluding infants born *via* caesarean section or born prematurely (Figure S1). Majority of the baseline characteristics of the infants and their parents were balanced between the GBS-exposed and GBS-unexposed groups. These characteristics, included infant sex, birth weight and length, feeding patterns in the first 8 months of life, parental height, weight, education level and employment status (Table S1). However, compared to those in the GBS-unexposed group, the GBS-exposed group exhibited a greater incidence of IAP use (*p* < 0.001), younger gestational age (39.4 vs. 39.8 weeks, *p* < 0.001) and older maternal age (30.0 vs. 29.2 years old, *p* = 0.047) at birth. These imbalanced factors were considered confounding variables in subsequent analyses of infant physical development.

In this study, we collected 323 eligible faecal samples from 205 infants aged 0–2 months, and 337 qualified vaginal samples from the third trimester for subsequent analysis.

### GBS-exposed infants exhibit a lower LAZ than GBS-unexposed infants

**Figure 1.**
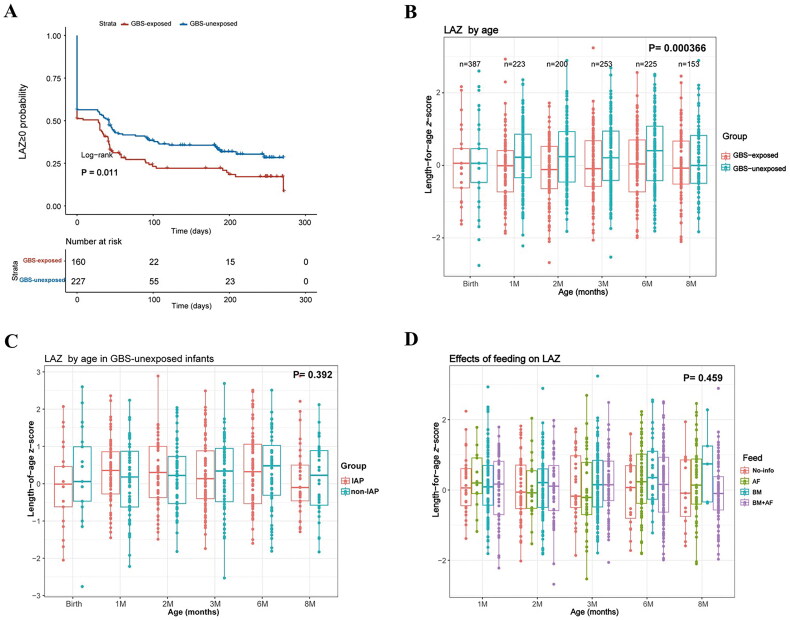
Impact of maternal GBS colonization during pregnancy on infant LAZ. (A) Kaplan–Meier curve depicting the occurrences of LAZ ≤ 0 in 387 infants, stratified by maternal GBS exposure and divided into 2 groups (GBS-exposed, and GBS-unexposed infants). P-value is displayed (log-rank test) without adjustment. (B) LAZ of GBS-exposed infants adjusted for maternal age, intrapartum antibiotic prophylaxis (IAP) and gestational age at delivery. (C, D) Effects of IAP(C) and feeding patterns (D) on infant LAZ after adjustment. The feeding patterns included artificial feeding (AF), breast feeding (BF), and mixed feeding.

To elucidate the effects of maternal GBS on infant physical growth, we initially analyzed LAZ, WAZ, WLZ, HCAZ and BMI *z*-score as measures of infant growth according to WHO child growth standards. Our observations revealed that, compared to those in the GBS-unexposed group, the infants in GBS-exposed group have a higher proportion of infants with LAZ < 0 within 8 months after birth (*p* = 0.011; Figure 1A) and show decreased LAZ (Figure 1B). The difference in the LAZ was most pronounced at 2 months of age (*p* = 0.002, Figure S2A). Importantly, even after adjusting for imbalanced confounders such as gestational age at birth, maternal IAP and age, the LAZ remained significantly lower in the GBS-exposed group than in the GBS-unexposed group during the first 8 months of life (*p* = 0.00037; Figure 1B).

Subsequently, we observed that the IAP had no significant impact on the LAZ in infants at any time point during the initial 8 months of life (Figure S2B). Furthermore, following adjustments for maternal GBS colonization, age and gestational age at delivery, the LAZ density remained similar between IAP infants and non-IAP infants in the GBS-unexposed group (Figure 1C). Additionally, we examined the impact of feeding patterns on infant LAZ, although feeding patterns were balanced between the two groups at every follow-up time (Table S1). After adjusting for maternal GBS, IAP, age and gestational age, we detected no significant differences in the LAZ between the various feeding patterns during the first 8 months of life (Figure 1D). These data suggest that the early decrease LAZ in GBS-exposed infants was caused by maternal GBS rather than by IAP or feeding patterns. Additionally, the GBS-exposed group exhibited a lower HCAZ than the GBS-unexposed group at 2 months of age (Figure S2C). However, after adjusting for confounders, the HCAZ, WAZ, WLZ and BMI *z-*score did not significantly differ between the two groups during the first 8 months of life (Figure S2D). Additionally, since calprotectin and secretory immunoglobulin A (sIgA) are involved in immune defence and resistance to pathogens [24], we tested their concentration and observed that neither maternal GBS nor IAP affected the levels of infant faecal calprotectin and sIgA during the first 3 months of life (Figure S3A-B). These findings suggest that maternal GBS is associated with infant LAZ and may contribute to adverse length growth. Therefore, we focused on the LAZ in infants, particularly at 2 months of age, when the LAZ was most significantly lower in the GBS-exposed group than in the GBS-unexposed group, and was independent of IAP or feeding patterns (Figure 1B-D, Figure S2A).

### Altered gut microbiota in GBS-exposed infants is associated with LAZ

**Figure 2.**
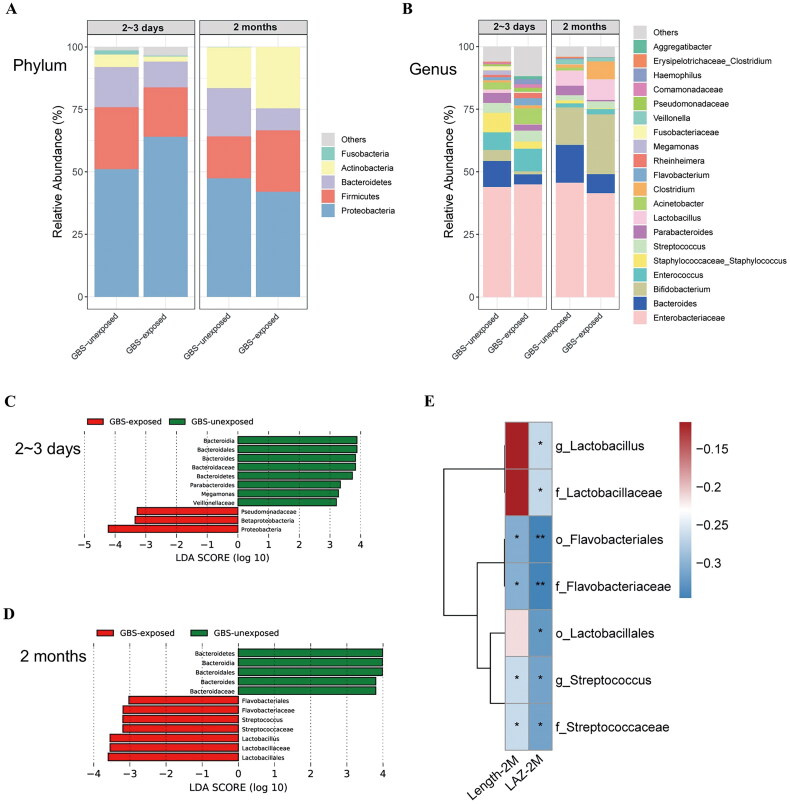
Relationship between altered gut microbiota in GBS-exposed infants and LAZ at 2–3 days and 2 months old. (A, B) Mean relative abundances of the 5 predominant phyla (A) and 20 predominant genera (B) in both groups at 2 time points. (C, D) Identification of differentially abundant bacterial taxa between the GBS-exposed and GBS-unexposed groups (|LDA| > 3.0) at 2–3 days(C) and 2 months (D). Red and green bars indicate taxa enriched in the GBS-exposed and GBS-unexposed groups, respectively. (E) Spearman correlation between differentially abundant bacterial taxa (|LDA|>2.0) and the LAZ at 2 months old (BH-adjusted). The correlation effect is indicated by a colour gradient from blue (negative correlation) to red (positive correlation). * *P* < 0.05, ** *P* < 0.01.

The gut microbiome plays a crucial role in early infant growth [15], and maternal health during pregnancy influences offspring growth by shaping the gut microbiome [25]. In this study, 16S rRNA gene sequence analysis revealed that at 2–3 days and 2 months of age, the GBS-exposed infants and the GBS-unexposed infants exhibited similar Shannon index, but the Jaccard distance was statistically different (Figure S4A-B). Specifically, the abundance of *Bacteroidetes* taxa, the second most common gut bacteria in early infancy, showed a broader reduction across levels from phylum to genus, encompassing *Bacteroidetes*, *Bacteroidia*, *Bacteroidales*, *Bacteroideceae* and *Bacteroides* (Figure 2A-C). Furthermore, its relative abundance at 2–3 days was positively correlated with infant HCAZ at 2 months (*p* < 0.01; Figure S4C). Additionally, at 2 months of age, the abundances of *Lactobacillus*, *Streptococcus* and *Flavobacteriaceae*, which were enriched in GBS-exposed infants, were negatively correlated with the LAZ (Figure 2B, D, E; Figure S4D).

### Altered gut metabolites in GBS-exposed infants are associated with LAZ

**Figure 3.**
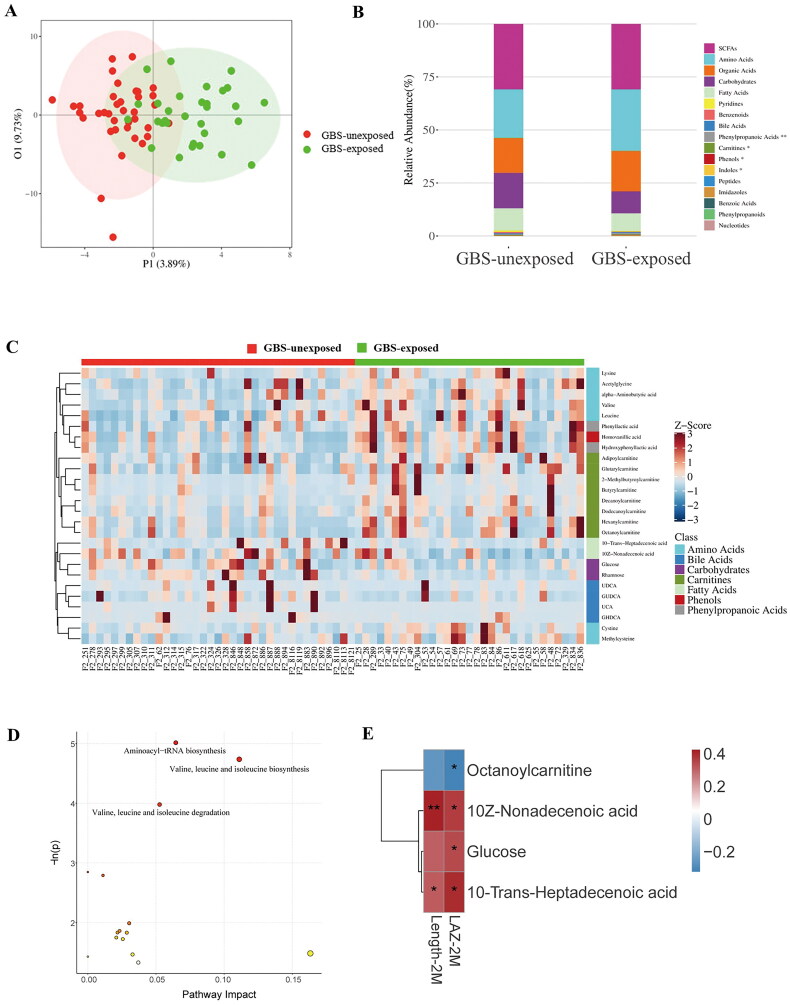
Association of altered gut metabolites in GBS-exposed infants with LAZ. (A-D) Orthogonal partial least squares discriminant analysis (OPLS-DA) (A), relative abundance of each metabolite class (B), different metabolites (C) and different metabolic pathways (D) in the two groups at 2 months of age. (E) Spearman correlation between gut metabolites and LAZ at 2 months (BH-adjusted). The correlation effect is indicated by a colour gradient from blue (negative correlation) to red (positive correlation). * *P* < 0.05, ** *P* < 0.01.

Subsequently, targeted metabolomics was used to measure 306 metabolites in 136 faecal samples from 70 infants. The basic characteristics and follow-up data of the two groups from the metabolomics analysis, including parental height and infant anthropometry at birth, were comparable to those of the entire cohort (Table S2). The analysis showed that gut metabolites in GBS-exposed infants underwent significant changes during the initial 2 months of life. Among the 229 metabolites detected, 1 increased and 16 decreased in GBS-exposed infants compared to GBS-unexposed infants at 2–3 days of age (Figure S5A-B). Additionally, at 2 months of age, 18 metabolites increased while 8 metabolites decreased (Figure 3A-C; Figure S5C; Table S3). Furthermore, at 2 months of age, these differentially abundant metabolites had an impact on the aminoacyl-tRNA biosynthesis pathway (*p* = 0.007; impact value = 0.065), as well as valine, leucine and isoleucine biosynthesis (*p* = 0.009; impact value = 0.111) and degradation (*p* = 0.019; impact value = 0.053) according to the pathway analysis conducted (Figure 3D). These data suggest that maternal GBS disrupts infant metabolites and metabolic functions.

Given that metabolites play a central role in the crosstalk between bacteria and hosts [26], we investigated the relationship between differentially abundant metabolites and infant growth at 2 months of age. Spearman correlation analysis revealed that the levels of decreased glucose, 10Z-nonadecenoic acid and 10-trans-heptadecenoic acid in GBS-exposed infants were positively correlated with LAZ, while the levels of increased octanoylcarnitine were negatively correlated with infant LAZ (Figure 3E, Table S3). Those findings support the notion that altered gut metabolites in GBS-exposed infants play a crucial role in influencing infant LAZ.

**Figure 4.**
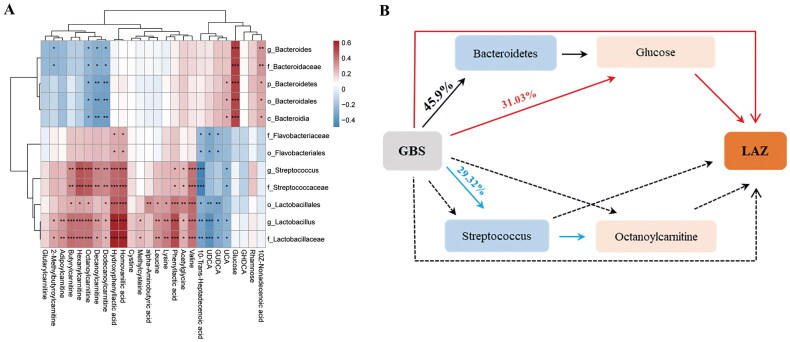
Mediation effect of altered microbiota and metabolites in GBS-exposed infants on the association between maternal GBS and infant LAZ at 2 months of age. (A) Spearman correlation between the altered bacteria and metabolites (BH-adjusted). The correlation effect is indicated by a colour gradient from blue (negative correlation) to red (positive correlation). *** *P* < 0.001, * *P* < 0.05, ** *P* < 0.01. (B) Mediation analysis of altered microbiota and metabolites in the association between maternal GBS and infant LAZ. Each line style represents a univariate mediation analysis (all results are provided in Table S4).

Subsequent analysis revealed a strong correlation between the gut microbiota and metabolites that exhibited significant differences between the two groups at 2 months of age. The abundances of *Bacteroidet*es and *Bacteroides* exhibited a positive correlation with 10Z-nonadecenoic acid and glucose, but displayed a negative correlation with octanoylcarnitine. Additionally, octanoylcarnitine was positively correlated with *Streptococcus* and *Lactobacillus*. Conversely, *Streptococcus*, *Lactobacillus* and *Flavobacteriaceae* were negatively correlated with 10-trans-heptadecenoic acid (Figure 4A).

Considering that GBS-exposed infants exhibited a lower LAZ, and that the LAZ was associated with an altered gut microbiota, we subsequently explored the contribution of modified microbiota and metabolites to maternal GBS-associated decreases in the LAZ. Mediation analysis revealed that the microbiota and metabolites associated with LAZ, which exhibited significant differences between the two groups, mediated the association between GBS exposure and decreased LAZ in infants at 2 months of age. In-depth analysis indicated that infant glucose tends to fully mediate infant LAZ (mediation effect of the b-estimate = 0.31026, 95% confidence interval [CI]= −0.00284 to 1.33, *p* = 0.056). *Bacteroidia* mediated 46.6% of the correlation between GBS and glucose (*p* =0.028), while *Streptococcus* mediated 29.32% of the correlation between GBS and octanoylcarnitine (*p* =0.032). Direct effects prevailed for the impacts of other different bacteria and metabolites on the correlation between GBS and LAZ (Table S4, Figure 4B). These findings suggest that the infant gut microbiota and metabolites mediate the impact of maternal GBS on infant LAZ.

### The neonatal microbiota, metabolites and maternal vaginal microbiota are associated with LAZ

**Figure 5.**
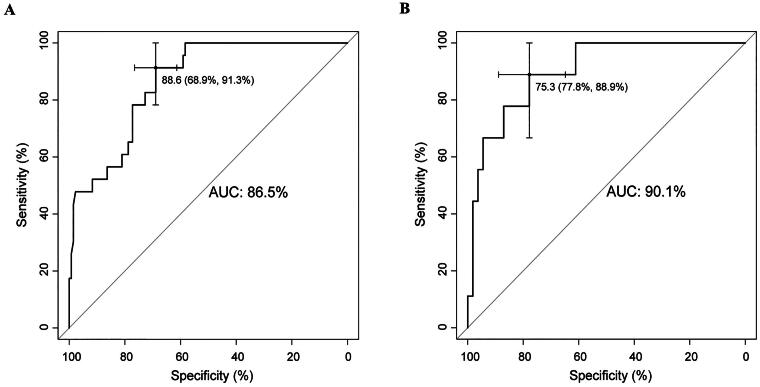
Associations between the gut microbiota, and metabolites levels in offspring aged 2–3 days and the occurrence of LAZ<-1 in infants aged 1-8 months. (A, B) The ROC curve depicts the identification of LAZ<-1 among all offspring aged 1-8 months. The data included baseline data, offspring data post birth, additional 16S rDNA data (A), and metabolite data (B) at 2–3 days of age. AUC, area under the curve.

Next, employing the least absolute shrinkage and selection operator (LASSO) approach, the Cox model, and proportional hazard assumption testing, we identified microbial taxa in infants aged 2–3 days. These taxa enhanced the discriminatory power (AUC = 0.865, C-index = 0.869) of the models for predicting the occurrence of LAZ<-1 in infants aged 1–8 months. Additionally, the metabolites also augmented the discriminatory power (AUC = 0.901, C-index = 0.883) over the baseline and birth data (Figure 5A-B, Figure S6C-E, Table S5). We subsequently classified all infants into three groups, Low1, Normal1 and High1, based on their overall LAZ from 1–8 months. These categories align with overall LAZ<-1, −1 ≤overall LAZ ≤ 1, and 1< overall LAZ. Subsequent analysis revealed distinctions in microbiota and metabolites between the Low1 and Normal1 groups at 2–3 days of age (Figure S6A, B). This observation implies that the gut microbiota and metabolites present during the initial days of life are linked to subsequent length growth and may contribute to reduced LAZ in GBS-exposed infants.

**Figure 6.**
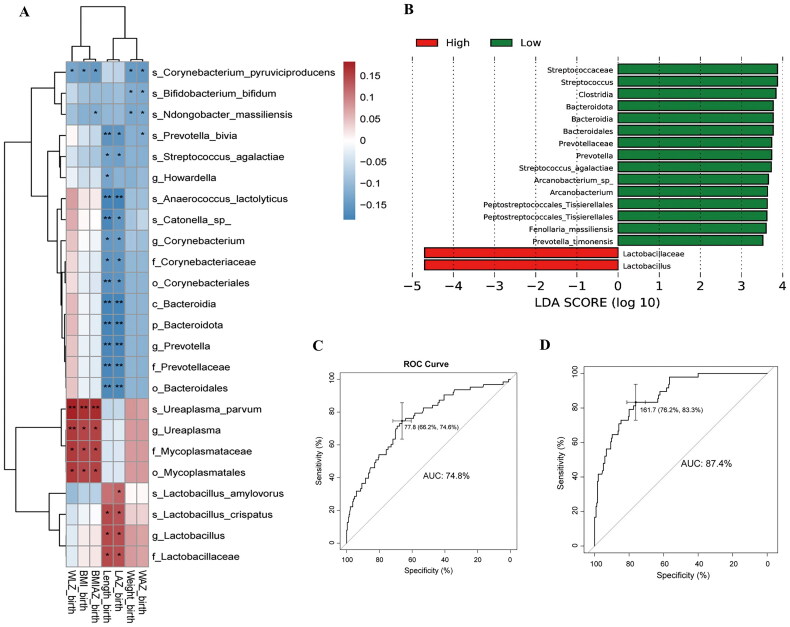
Association between the vaginal microbiota of the GBS group in the third trimester and infant length. (A) Spearman correlations between infant growth at birth and altered vaginal bacterial taxa of the GBS group in the third trimester (BH-adjusted). The colour gradient represents the correlation effect, ranging from blue (negative correlation) to red (positive correlation). * *P* < 0.05, ** *P* < 0.01. (B) The difference of vaginal bacterial taxa in third trimester between the High and Low groups (|LDA| > 3.5). High and Low refer to infants with 1< overall LAZ and overall LAZ<-1, respectively, in the age range of 0-8 months. Red, and green bars indicate bacterial taxa enriched in the High and Low groups, respectively. (C, D) ROC curve displaying the discriminatory power of LAZ<-1 in infants aged 0-8 months using baseline data and paired maternal vaginal microbiota in the third trimester (C), and in infants aged 1-8 months using baseline data, paired maternal vaginal microbiome in the third trimester, and offspring data after birth (D). AUC, area under the curve.

Here, we aimed to explore the correlation between maternal vaginal microbiota in the third trimester and decreased LAZ in GBS-exposed infants. Initially, 16S rRNA gene sequencing demonstrated distinct microbiota compositions in the GBS group compared to those in the healthy group, characterized by elevated GBS and reduced *Lactobacillus crispatus* (Figure S7A-B). Distinct bacterial taxa (|LDA|>2) are associated with infant growth at birth, particularly in terms of length growth (Figure 6A). Subsequently, mothers were categorized into three groups based on the overall LAZ of their infant during the 0–8 months period: Low (overall LAZ<-1), Normal (-1 ≤ overall LAZ ≤ 1) and High (1 < overall LAZ). The analysis revealed significant differences in the vaginal microbiota between the Low and Normal groups, as well as between the Low and High groups (Figure S7C-D). Additionally, the Low group exhibited enrichment of GBS, suggesting a correlation between maternal vaginal GBS during pregnancy and infant length growth (Figure 6B). Finally, we evaluated the discriminatory power of the models utilizing third-trimester vaginal microbiota data to predict the occurrence of LAZ<-1 in early infancy. As anticipated, vaginal bacteria exhibited enhanced discriminatory power with an AUC of 74.8 (C-index = 0.712) for predicting the occurrence of LAZ<-1 in infants aged 0–8 months over baseline data (Figure 6C, Figure S7E, Table S5) and an AUC of 0.874 (C-index = 0.868) for predicting age 1–8 months over baseline and birth data (Figure 6D, Figure S6C-E, Figure S7F, Table S6). These findings suggest an association between maternal GBS and lower LAZ in infants, possibly mediated by alterations in the vaginal microbiota.

## Discussion

Our comprehensive analysis of 1441 measurements of growth during the first 8 months of life revealed a decrease in the LAZ in GBS-exposed infants compared to that in their GBS-unexposed counterparts. This discrepancy was most notable at 2 months of age, providing support for the hypothesis that maternal GBS during pregnancy may adversely affect the length growth of offspring in early life. Faecal multi-omics analysis of 205 infants revealed alterations in the gut microbiota and metabolites in GBS-exposed infants. These alterations correlated with growth at 2 months of age and mediated the adverse effects of maternal GBS on the LAZ in offspring. Expanding our investigation to include 337 vaginal samples, we observed that the vaginal microbiota of GBS-colonized women was altered in the third trimester and correlated with infant growth. Integration of all the datasets revealed distinct differences in maternal vaginal microbiota, infant gut microbiota and metabolites between infants with overall LAZ<-1 and their counterparts. This collective insight significantly enhanced the discriminatory power of the baseline data, enabling more accurate predictions of the occurrence of LAZ<-1 during the first 8 months of life.

Our investigation revealed that maternal GBS colonization during pregnancy has the potential to hinder infant length growth by disrupting maternal vaginal microbiota, infant gut microbiota and metabolites. The GBS-exposed group is characterized by a younger gestational age and an older maternal age, but prior studies suggest that small differences in gestational age (less than 1 week) and maternal age (more than 1 year) lack biologically relevant [27,28]. GBS or IAP exposure does not influence infant faecal immunity during the first 3 months of life.

Notably, recent clinical trials have highlighted the impact of maternal factors, such as gestational diabetes mellitus and HIV infection, on infant weight gain by influencing the composition of the infant gut microbiota [25,29,30]. The observed lower LAZ in GBS-exposed infants in our study supports the notion that maternal GBS during pregnancy may indeed contribute to the delay in infant length growth. Decreased *Bacteroidetes* and glucose in infants exposed to GBS exhibited a positive correlation, concurrently demonstrating a positive association with length growth. These interrelations further served to mediate the impact of maternal GBS on infant LAZ. This alignment of factors is particularly intriguing in light of previous reports linking decreased *Bacteroidetes* levels to child overweight [31]. This association is likely attributed to the role of *Bacteroides* in the later stages of gut microbiota maturation [32,33], where it has emerged as a pivotal contributor to polysaccharide utilization [34,35]. Notably, a reduction in *Bacteroidetes* has been shown to influence skeletal maturation through the gut-liver axis in murine models [36]. Additionally, as the primary energy source for all living organisms, glucose participates in metabolic pathways including glycolysis and gluconeogenesis, as well as metabolic homeostasis promoting osteogenesis and skeletal development [37–39]. Our data revealed a decrease in the abundance of vaginal *Lactobacillus* in women colonized with GBS, a phenomenon that was positively correlated with the overall LAZ of infants. Intriguingly, *Lactobacillus* was found to be enriched in the faecal of GBS-exposed infants, and exhibited a negative association with the LAZ at 2 months of age. These dynamic findings suggest that, in response to reduced glucose and compromised growth in infants, *Lactobacillus* may accelerate intestinal maturation [40], enhance nutrient sensing, mediate glucose homeostasis [41], and ultimately promote infant growth. Furthermore, we not only demonstrated a positive correlation between increased levels of octanoylcarnitine and *Streptococcus* in infants exposed to GBS but also revealed a negative correlation with growth. Intriguingly, these factors mediated the impact of maternal GBS on infant LAZ, consistent with previously reported negative correlations between octanoylcarnitine and LAZ [42,43]. Ultimately, we observed significant increases in *Streptococcus* and *Streptococcus agalactiae* in the vagina of GBS-positive women, which were inversely associated with overall LAZ in infants during the initial 8 months of life. Complemented by the decreased LAZ observed in GBS-exposed infants, we postulate that this phenomenon may be attributed to the shaping of gut microbial assembly in naturally born infants by the maternal vaginal microbiota [19,23,44–46]. This intricate interplay may further impact offspring metabolism, immunity and growth [20]. In conclusion, our findings propose that maternal GBS might decrease offspring length growth by influencing the infant microbiota and metabolites. Furthermore, the role of the vaginal microbiota during pregnancy appears to be integral to this process. However, the precise mechanisms underlying these occurrences remain to be determined.

IAP and perinatal antibiotic exposure have been associated with infant weight [47,48] and neurobehavior [49]. These associations may signify intricate metabolic changes [50], impacts on immunity, alterations in the gut microbiota [51,52], and nuances in neurodevelopment [53]. Conversely, conflicting evidence has emerged, indicating that IAP administered specifically for GBS is not associated with a higher childhood BMI [9]. Considering the high proportion of IAP used in GBS-unexposed women to prevent perinatal infection, we note an inherent disparity in IAP administration between GBS-exposed and GBS-unexposed groups. This disparity mirrors the essential medical practices for GBS-positive pregnant women who follow clinical practice guidelines to prevent maternal and infant infections [54]. And our study aims to investigate the potential impact of IAP on infant LAZ. Intriguingly, our unexpected findings indicate that, while maternal GBS significantly influences infant LAZ in the first 8 months of life, IAP itself does not emerge as a substantial factor. Consequently, our study primarily delves into the influence of maternal GBS on infant LAZ and comprehensively analyses the interconnections among the maternal vaginal microbiome, infant gut microbiota, metabolism and LAZ.

The strengths of this study are twofold. First, an estimated 19.3 million infants were exposed to maternal GBS in 2020 without developing invasive GBS disease due to IAP [7]. However, there is a notable absence of evidence exploring the impact of maternal GBS on infant growth. This study is pioneering in revealing adverse length growth in infants exposed to maternal GBS in early life, providing valuable insights into the growth patterns of these infants. Second, acknowledging the crucial roles of the gut microbiome and metabolomics in infant physical growth [20,21]. This study demonstrated the intricate interplay among the infant gut microbiota, metabolites and maternal vaginal microbiota may contribute to the diminished length growth observed in GBS-exposed infants. And it also offers evidence that interventions targeting both maternal vaginal microbiota and infant microbiota or metabolites may enable these infants to achieve height growth comparable to that of GBS-unexposed infants.

However, there are several additional limitations. First, this study was conducted in southern China over an 8-month follow-up period. The conclusions regarding metabolites are derived from a limited subset of participants. Additionally, the reason for non-statistical difference in LAZ after 2 months old between the GBS-exposed infants and GBS-exposed infants remain unclear. Thus, cohort studies with broader geographical contexts, larger sample sizes and extended follow-up durations are imperative to validate our findings. Second, the precise mechanisms underlying the effects of maternal GBS on infant length growth need further investigation. Finally, considering that GBS may lead to severe infections in both mothers and infants, a randomized controlled trial is not a cost-effective approach to elucidate the effects of GBS or IAP on offspring growth. Nevertheless, more appropriate methods to address this concern are necessary in future research.

## Conclusions

Our study suggested that maternal GBS may adversely affect infant length growth by influencing the infant’s gut microbiome and metabolites, with potential implications linked to the maternal vaginal microbiome. However, the extensive and long-term investigations are paramount for fully comprehending the effects of maternal GBS on infant growth, with a specific focus on the interplay among maternal vaginal microbiota, the infant microbiome and metabolites in this dynamic process.

## Supplementary Material

Supplemental Material

## Data Availability

The 16S rRNA sequencing data supporting the findings of this study are available in the NCBI BioProject database at https://www.ncbi.nlm.nih.gov/sra/PRJNA943482 (PRJNA943482). The targeted metabolomics data are available in the MetaboLights database at https://www.ebi.ac.uk/metabolights (MTBLS7419). All the other data in this study are available from the corresponding author, Q. Wang, upon reasonable request.
