# Supplementary material for: Maternal group B Streptococcus decreases infant length and alters the early-life microbiome: a prospective cohort study
Source: Ann Med. 2024 Dec 18;57(1):2442070. doi: 10.1080/07853890.2024.2442070 (PMC11656753; doi:10.1080/07853890.2024.2442070)
Supplement: Supplemental Material [file IANN_A_2442070_SM4854.zip › suppl_data/Supplementary_materials_248084795.docx]

**Supplement materials**

**Supplementary Methods:**

**Method S1:** **16S rRNA gene sequence and analysis**

**Method S2: Targeted metabolomics analyses**

**Supplementary Figures:**

**Figure S1. Flow diagram illustrating the study protocol.**

**Figure S2. Comparison of gut immunity in infants exposed to maternal GBS or IAP.**

**Figure S3.** **Impact of maternal GBS on infant growth.**

**Figure S4.** **Association of altered gut microbiota in GBS-exposed infants with infant growth.**

**Figure S5.** **Altered gut metabolites in GBS-exposed infants at 2-3 days of age.**

**Figure S6.** **The gut microbiota and metabolome of offspring aged 2-3 days** **and their ability to identify the occurrence of LAZ<-1 in offspring aged 1-8 months.**

**Figure S7.** **Association of altered vaginal microbiota in the GBS group during the third trimester with overall LAZ<-1 in infants aged 0-8 months.**

**Supplemental Methods**

**Method S1:** **16S rRNA gene sequence and analysis**

For fecal samples (n=279; 2-3 days, 180; 2 months, 99), 16S rRNA gene V4 region was amplified using barcoded primers 515F (GTGYCAGCMGCCGCGGTAA) and 806R (GGACTACNVGGGTWTCTAAT) with Applied Biosystems ViiA 7 Real-Time PCR System (Thermo Fisher Scientific, USA). Vaginal samples (n=337), underwent amplification of the 16S rRNA gene V1-V3 region using a mixture of barcoded primers 27F and 533R (ATTACCGCGGCTGCTGG) to maximize sequence type discovery and eliminate the PCR amplification bias. The 27F primer mixture comprised four-fold 27f-A (5'-AGAGTTYGATYMTGGCTYAG) and one-fold 27f-B (AGARTTTGATCYTGGTTCAG).

For the feces, amplification followed the procedures outlined in our previous study,^[1]^ including initial denaturation at 94 °C for 5 min, followed by 30 cycles of denaturation at 94 °C for 30 s, annealing at 52 °C for 30 s, extension at 72 °C for 45 seconds, and a final extension at 72 °C for 5 min. Amplification for vaginal samples included initial denaturation at 95 °C for 5 min, followed by 29 cycles of denaturation at 94 °C for 30 s, annealing at 52 °C for 30 s, extension at 72 °C for 1 min, and a final extension at 72 °C for 10 min. All samples underwent randomization at the PCR stage and again at the sequencing stage, and were sequenced with the Illumina NovaSeq sequencer (2×250 bp paired-end).

The raw sequence data were demultiplexed into paired-end fastq files based on unique barcodes and truncated by cutting off the barcodes and primer sequences using a customized Perl script. For the gut samples, the reads were processed using the DADA2 denoise-paired plugin (Version 2021.4) in QIIME2 (Version 2021.4) for denoising, including the following steps: quality filtering, dereplication and chimeras filtering with the ASV (amplicon sequence variant) table created. The sequences in the ASV table were annotated in QIIME2 using the pre-fitted scikit-learn-based taxonomy classifier based on the Greengenes (Version 138). For vaginal samples, forward sequences were imported to QIIME2 and processed using the DADA2 denoise-single plugin (Version 2021.4) for denoising, including the following steps: quality filtering, dereplication, and chimeras filtering with the ASV table created. The sequences in the ASV table were annotated in QIIME2 using the prefitted scikit-learn-based taxonomy classifier based on the combination of SILVA (Version 138) and RDP (Release 11.5).

β-diversity analysis was conducted in QIIME2 to evaluate dissimilarities in microbial communities between groups, visualized through principal-coordinate analysis (PCoA), and statistically validated via permutational multivariate analysis of variance (PERMANOVA). Linear discriminant analysis effect size (LEfSe) analysis was used to detect the differential microbial features between groups, of which the |LDA| scores (log10) > 2.0.

Relative abundance of bacterial genera was statistically compared between groups using Wilcoxon rank-sum test. Data visualization and statistical analyses were performed with ggplot2 (Version 3.3.5) in Rstudio (Version 1.4.1106) under R (Version 4.1.1)^[2]^.

**Method S2:** Targeted metabolomics analyses

Targeted metabolomics of fecal samples was performed by Metabo-Profile (Shanghai, China). quantifying 229 metabolites across 17 classes in 136 fecal samples. Detailed measurement information as following description.

Freeze-dried feces (5 mg) were homogenized with 25 μL of ultrapure water, and metabolites were extracted with 120 μL of methanol containing internal standard. After centrifugation, 20 μL of supernatant underwent derivatization with 20 μL of freshly derived reagents on an Eppendorf epMotion Workstation (Eppendorf Inc., Hamburg, Germany). Subsequently, 330 μL of ice-cold 50% methanol solution was added to dilute the derivatized sample. After another centrifugation, 135 μL of supernatant was mixed with 10 μL of internal standards. Finally, derivatized samples and serial dilutions of derivatized stock standards were analyzed and quantitated by ultra-performance liquid chromatography coupled to tandem mass spectrometry (UPLC‒MS/MS) (ACQUITY UPLC-Xevo TQ-S, Waters Corp., Milford, MA, USA).

Quality control involved three types of samples: test mixtures, internal standards, and pooled biological samples, ensuring high-quality analytical results. In analyzsis of the metabolomes of 136 fecal samples, 13 quality control samples prepared by mixing derivatized samples were analyzed throughout the analytical run.

The raw data files generated by UPLC‒MS/MS were processed using TMBQ software (v1.0, Metabo-Profile, Shanghai, China) and analyzed with the iMAP (v1.0, Metabo-Profile, Shanghai, China) platform.

**References**

[1] LI J, WANG H, QING W, et al. Congenitally underdeveloped intestine drives autism-related gut microbiota and behavior [J]. Brain, behavior, and immunity, 2022, 105: 15-26.

[2] YANG Y H, LI D L, BI X Y, et al. Acetylcholine Inhibits LPS-Induced MMP-9 Production and Cell Migration via the α7 nAChR-JAK2/STAT3 Pathway in RAW264.7 Cells [J]. Cellular physiology and biochemistry : international journal of experimental cellular physiology, biochemistry, and pharmacology, 2015, 36(5): 2025-38.
