# Supplementary material for: Maternal group B Streptococcus decreases infant length and alters the early-life microbiome: a prospective cohort study
Source: Ann Med. 2024 Dec 18;57(1):2442070. doi: 10.1080/07853890.2024.2442070 (PMC11656753; doi:10.1080/07853890.2024.2442070)
Supplement: Supplemental Material [file IANN_A_2442070_SM4854.zip › suppl_data/Supplement_Tables.docx]

**Supplemental tables:**

**Table S1.** Comparison of parental and infant characteristics between the GBS-exposed and GBS-unexposed groups

**Table S2.** Characteristics of GBS-exposed and GBS-unexposed infants for metabolomics analysis

**Table S3.** Differential metabolites between GBS-exposed and GBS-unexposed groups at 2 months of age

**Table S4.** Mediation analysis of microbiota and metabolites in the effect of maternal GBS on infants’ LAZ

**Table S5.** Markers and discrimination for distinguishing LAZ<-1 in infants aged 1-8 months selected by LASSSO

**Table S6.** Markers and discrimination for distinguishing LAZ<-1 in infants aged 0-8 months selected by LASSO

**Table S1.** Comparison of parental and infant characteristics between the GBS-exposed and GBS-unexposed groups

| **Variables** | ***N*** | **All** | **GBS-unexposed** | **GBS-exposed** | ***P* value** |
| --- | --- | --- | --- | --- | --- |
|  |  |  | **N=227** | **N=160** |  |
| Cities, *n* (%) | 387 |  |  |  | 0.651 |
| GZ |  | 187 (48.3) | 107 (47.1) | 80 (50.0) |  |
| ZS |  | 200 (51.7) | 120 (52.9) | 80 (50.0) |  |
| **Maternal** |  |  |  |  |  |
| Age, mean (SD), years | 387 | 29.6 (3.95) | 29.2 (3.90) | 30.0 (3.99) | 0.047 |
| Employed, *n* (%) | 387 | 322 (83.2) | 187 (82.4) | 135 (84.4) | 0.705 |
| Nationality, *n* (%) | 387 |  |  |  | 0.17 |
| Gaeml |  | 1 (0.26) | 0 (0.00) | 1 (0.62) |  |
| Han nationality |  | 385 (99.5) | 227 (100) | 158 (98.8) |  |
| Zhuang |  | 1 (0.26) | 0 (0.00) | 1 (0.62) |  |
| Gestational age, mean (SD), w | 387 | 39.7 (0.91) | 39.8 (0.85) | 39.4 (0.94) | <0.001 |
| IAP, n (%) | 387 | 234 (60.5) | 98 (43.2) | 136 (85.0) | <0.001 |
| Height, mean (SD), cm | 387 | 160 (5.30) | 160 (5.57) | 159 (4.86) | 0.111 |
| Weight before delivery, mean (SD), kg | 387 | 66.2 (8.87) | 66.2 (8.62) | 66.1 (9.24) | 0.849 |
| BMI before delivery, mean (SD) | 387 | 25.9 (2.95) | 25.9 (2.78) | 26.1 (3.18) | 0.506 |
| Hospitalization time after delivery, mean (SD), days | 387 | 2.16 (0.88) | 2.19 (0.64) | 2.11 (1.14) | 0.406 |
| Education, *n* (%) | 387 |  |  |  | 0.434 |
| Primary/Junior secondary |  | 34 (8.79) | 23 (10.1) | 11 (6.88) |  |
| Senior secondary |  | 94 (24.3) | 57 (25.1) | 37 (23.1) |  |
| Higher |  | 259 (66.9) | 147 (64.8) | 112 (70.0) |  |
| **Paternal** |  |  |  |  |  |
| Age, mean (SD), years | 364 | 31.0 (4.76) | 30.6 (4.46) | 31.6 (5.09) | 0.05 |
| Employed, *n* (%) | 387 | 385 (99.5) | 227 (100) | 158 (98.8) | 0.17 |
| Height, mean (SD), cm | 204 | 172 (5.90) | 172 (5.73) | 173 (6.21) | 0.783 |
| Weight, mean (SD), kg | 204 | 69.2 (9.12) | 69.3 (9.64) | 69.0 (8.23) | 0.816 |
| BMI, mean (SD) | 204 | 23.3 (2.63) | 23.3 (2.81) | 23.1 (2.30) | 0.649 |
| Education, *n* (%) | 387 |  |  |  | 0.454 |
| Primary/Junior secondary |  | 27 (6.98) | 17 (7.49) | 10 (6.25) |  |
| Senior secondary |  | 83 (21.4) | 53 (23.3) | 30 (18.8) |  |
| Higher |  | 277 (71.6) | 157 (69.2) | 120 (75.0) |  |
| Household incomes, mean (SD), w/year | 181 | 25.8 (12.7) | 25.0 (12.7) | 27.3 (12.7) | 0.236 |
| **Infant** |  |  |  |  |  |
| Sex, female, *n* (%) | 387 | 196 (50.6) | 122 (53.7) | 74 (46.2) | 0.177 |
| Apgar, mean (SD) | 387 |  |  |  |  |
| 1 min |  | 9.97 (0.19) | 9.98 (0.20) | 9.97 (0.17) | 0.629 |
| 5 min |  | 10.00 (0.05) | 10.00 (0.07) | 10.0 (0.00) | 0.318 |
| 10 min |  | 10.00 (0.05) | 10.00 (0.07) | 10.0 (0.00) | 0.318 |
| Premature delivery, *n* (%) | 387 |  | 0 (0.00) | 1 (0.63) |  |
| Birth weight, mean (SD), g | 387 | 3224 (357) | 3252 (348) | 3185 (366) | 0.068 |
| Birth length, mean (SD), cm | 387 | 49.6 (1.57) | 49.7 (1.62) | 49.4 (1.49) | 0.131 |
| Length-for-age *z-*score at birth, mean (SD), | 387 | 0.04 (0.84) | 0.10 (0.85) | -0.06 (0.81) | 0.065 |
| Total NICU, *n* (%) | 387 | 68 (17.6) | 39 (17.2) | 29 (18.1) | 0.917 |
| **Feeding patterns** |  |  |  |  |  |
| 1 month, *n* (%) | 274 |  |  |  | 0.754 |
| AF |  | 26 (9.49) | 15 (9.62) | 11 (9.32) |  |
| BM |  | 177 (64.6) | 98 (62.8) | 79 (66.9) |  |
| BM+AF |  | 71 (25.9) | 43 (27.6) | 28 (23.7) |  |
| 2 months, *n* (%) | 274 |  |  |  | 0.838 |
| AF |  | 30 (10.9) | 16 (10.3) | 14 (11.9) |  |
| BM |  | 163 (59.5) | 92 (59.0) | 71 (60.2) |  |
| BM+AF |  | 81 (29.6) | 48 (30.8) | 33 (28.0) |  |
| 3 months, *n* (%) | 228 |  |  |  | 0.358 |
| AF |  | 41 (18.0) | 23 (17.2) | 18 (19.1) |  |
| BM |  | 124 (54.4) | 78 (58.2) | 46 (48.9) |  |
| BM+AF |  | 63 (27.6) | 33 (24.6) | 30 (31.9) |  |
| 6 months, *n* (%) | 203 |  |  |  | 0.131 |
| AF |  | 62 (30.5) | 28 (24.8) | 34 (37.8) |  |
| BM |  | 31 (15.3) | 18 (15.9) | 13 (14.4) |  |
| BM+AF |  | 110 (54.2) | 67 (59.3) | 43 (47.8) |  |
| 8 months, *n* (%) | 138 |  |  |  | 0.163 |
| AF |  | 51 (37.0) | 19 (30.6) | 32 (42.1) |  |
| BM |  | 5 (3.62) | 1 (1.61) | 4 (5.26) |  |
| BM+AF |  | 82 (59.4) | 42 (67.7) | 40 (52.6) |  |

*Note*. *N* or *n*, number of participants; w, weeks; SD, standard deviation; cm, centimeter; kg, kilogram; k, gram. AF, Artificial feeding; BF, Breastfeeding; BM+AF, Mixed Feeding.

For normally distributed variables, ANOVA or *t*-test was employed. For non-normally distributed variables, Kruskal and Benjamini-Hochberg adjustment were utilized, and for categorical variables, chi-square tests or Fisher’s exact test were employed to calculate statistical significance. The Shapiro-Wilks test was applied to detect normal distribution.

**Table S2.** Characteristics of GBS-exposed and GBS-unexposed infants for metabolomics analysis

| **Characteristics** | ***N*** | **All** | **GBS-unexposed** | **GBS-exposed** | ***P* value** |
| --- | --- | --- | --- | --- | --- |
|  |  |  | **N=37** | **N=33** |  |
| IAP, *n* (%) | 70 | 36（51.4%） | 10 (27.0%) | 26 (78.8%) | <0.001 |
| Gender, female, *n* (%) | 70 | 35（50%） | 17 (45.9%) | 18 (54.5%) | 0.632 |
| Gestational age, mean (SD), days | 70 | 277（6.83） | 279 (5.75) | 275 (7.11) | 0.003 |
| Total NICU, *n* (%) | 70 | 7（10%） | 6 (16.2%) | 1 (3.03%) | 0.11 |
| Age-mother, mean (SD), years | 70 | 30.2（4.30） | 29.8 (3.94) | 30.7 (4.69) | 0.415 |
| Nationality: Han nationality, *n* (%) | 70 | 70（100%） | 37 (100%) | 33 (100%) | . |
| Employed-mother, *n* (%) | 70 | 62（88.6%） | 34 (91.9%) | 28 (84.8%) | 0.462 |
| Education-mother: |  |  |  |  | 0.216 |
| Higher | 70 | 54（77.1%） | 31 (83.8%) | 23 (69.7%) |  |
| Primary/Junior secondary | 70 | 5（7.14%） | 1 (2.70%) | 4 (12.1%) |  |
| Senior secondary | 70 | 11（15.7%） | 5 (13.5%) | 6 (18.2%) |  |
| Hight-mother, mean (SD), cm | 70 | 160(5.40) | 160 (5.45) | 159 (5.39) | 0.603 |
| Weight-mother, mean (SD), kg | 70 | 60.5(8.2) | 64.4 (8.64) | 65.7 (7.74) | 0.517 |
| BMI-mother, mean (SD) | 70 | 25.5(2.80) | 25.1 (2.41) | 25.9 (3.16) | 0.235 |
| Age-father, mean (SD), years | 70 | 31.5（5.55） | 30.2 (5.15) | 33.1 (5.67) | 0.031 |
| Height-father, mean (SD), cm | 69 | 173(5.95) | 174 (6.19) | 173 (5.73) | 0.598 |
| Weight-father, mean (SD), kg | 69 | 69.5(7.96) | 69.4 (8.34) | 69.6 (7.64) | 0.909 |
| BMI-father, mean (SD) | 69 | 23.1（2.26） | 23.0 (2.39) | 23.3 (2.14) | 0.627 |
| Education-father: |  |  |  |  | 0.606 |
| Higher | 70 | 53（75.7%） | 30 (81.1%) | 23 (69.7%) |  |
| Primary/Junior secondary | 70 | 7（10.0%） | 3 (8.11%) | 4 (12.1%) |  |
| Senior secondary | 70 | 10（14.3%） | 4 (10.8%) | 6 (18.2%) |  |
| Household incomes, mean es, mean (SD), w/year | 66 | 27.9（10.4） | 26.8 (10.5) | 29.1 (10.3) | 0.365 |
| Apgar-1min, mean (SD) | 70 | 9.99（0.12） | 10.0 (0.00) | 9.97 (0.17) | 0.325 |
| Length- birth, mean (SD), cm | 70 | 49.8（1.33） | 49.8 (1.41) | 49.8 (1.25) | 0.868 |
| LAZ-birth, mean (SD) | 70 | 0.14（0.71） | 0.14 (0.73) | 0.14 (0.70) | 0.987 |
| birth weight, mean (SD), kg | 70 | 3.22（0.33） | 3.28 (0.33) | 3.16 (0.32) | 0.125 |
| WAZ-birth, mean (SD) | 70 | -0.16（0.71） | -0.05 (0.70) | -0.28 (0.71) | 0.166 |
| BMI-birth, mean (SD) | 70 | 13.0（1.23） | 13.2 (1.19) | 12.8 (1.24) | 0.124 |
| BMIAZ-birth, mean (SD) | 70 | -0.33（0.99） | -0.14 (0.93) | -0.55 (1.03) | 0.087 |
| WLZ-birth, mean (SD) | 70 | -0.33（1.13） | -0.15 (1.08) | -0.54 (1.15) | 0.149 |

*Note*. *N* or *n*, number of participants; SD, standard deviation; cm, centimeter; kg, kilogram.

For normally distributed variables, ANOVA or *t*-test was employed; for non-normally distributed variables, Kruskal and Benjamini-Hochberg adjustment were applied; and for categorical variables, chi-square tests or Fisher’s exact test were utilized to calculate statistical significance. The Shapiro-Wilks test was used to detect normal distribution.

**Table S3.** Differential metabolites between GBS-exposed and GBS-unexposed groups at 2 months of age

| **Class** | **Metabolite** | ***P* value** | **log2FC** | **OPLSDA_VIP** |
| --- | --- | --- | --- | --- |
| Amino Acids | Lysine | 0.043 | 1.453 | 1.519 |
| Amino Acids | Cystine | 0.017 | 0.454 | 2.300 |
| Amino Acids | Methylcysteine | 0.021 | 0.515 | 2.146 |
| Phenylpropanoic Acids | Hydroxyphenyllactic acid | 0.004 | 1.512 | 1.820 |
| Amino Acids | alpha-Aminobutyric acid | 0.011 | 1.602 | 1.315 |
| Amino Acids | Acetylglycine | 0.023 | 0.954 | 0.720 |
| Amino Acids | Valine | 0.033 | 1.068 | 1.310 |
| Carbohydrates | Glucose | 0.033 | -0.799 | 2.078 |
| Amino Acids | Leucine | 0.050 | 0.569 | 1.782 |
| Carbohydrates | Rhamnose | 0.027 | -0.867 | 1.922 |
| Phenols | Homovanillic acid | 0.006 | 1.724 | 2.005 |
| Phenylpropanoic Acids | Phenyllactic acid | 0.029 | 0.988 | 1.526 |
| Bile Acids | GUDCA | 0.029 | -1.702 | 1.716 |
| Bile Acids | GHDCA | 0.026 | -1.048 | 0.430 |
| Bile Acids | UDCA | 0.011 | -1.279 | 1.045 |
| Fatty Acids | 10-Trans-Heptadecenoic acid | 0.024 | -1.072 | 1.980 |
| Fatty Acids | 10Z-Nonadecenoic acid | 0.018 | -0.611 | 1.218 |
| Carnitines | Butyrylcarnitine | 0.021 | 1.198 | 2.195 |
| Carnitines | 2-Methylbutyroylcarnitine | 0.037 | 1.181 | 1.855 |
| Carnitines | Glutarylcarnitine | 0.024 | 0.773 | 2.106 |
| Carnitines | Hexanylcarnitine | 0.019 | 3.511 | 2.147 |
| Carnitines | Adipoylcarnitine | 0.027 | 0.481 | 1.160 |
| Carnitines | Octanoylcarnitine | 0.002 | 1.595 | 2.472 |
| Carnitines | Decanoylcarnitine | 0.007 | 2.378 | 2.269 |
| Carnitines | Dodecanoylcarnitine | 0.014 | 0.988 | 2.395 |
| Bile Acids | UCA | 0.033 | -1.103 | 1.680 |

*Note*. log2FC, fold change of log2 of differential metabolites in the GBS-exposed group relative to the GBS-unexposed group; OPLSDA_VIP, Variable Important in Projection by Orthogonal Partial Least Squares Discriminant Analysis; GUDCA, glycoursodeoxycholic acid; GHDCA, glycohyodeoxycholate; UDCA, ursodeoxycholic acid; UCA, ursocholic acid.

**Table S4.** Mediation analysis of microbiota and metabolites in the effect of maternal GBS on infants’ LAZ

| **X** | **M** | **Y** | **Total Effect** | **ADE（95%CI，*P*）** | **ACME（95%CI，*P*）** | **ACME proportion** | **Proportion, Mediated** |
| --- | --- | --- | --- | --- | --- | --- | --- |
| GBS | Bacteroidetes | Glucose | 17400 (2.93e+03~33015.81, 0.030*) | 9200 (-5.10e+03~23900.26, 0.176) | 8220 (7.64e+02~18223.01, 0.038*) | 0.4724 | 45.9% (-1.28e-02~1.71, 0.060.) |
| GBS | Bacteroidia | Glucose | 17900 (2.83e+03~33107.22, 0.020*) | 9410 (-4.17e+03~22718.06, 0.172) | 8530 (8.71e+02~17393.55, 0.028*) | 0.4765 | 46.6% (5.44e-02~1.59, 0.028*) |
| GBS | Bacteroidales | Glucose | 17700 (9.80e+02~33627.05, 0.034*) | 9320 (-4.29e+03~24008.35, 0.216) | 8340 (9.88e+02~17087.18, 0.032*) | 0.4712 | 46.1% (2.73e-02~1.59, 0.050*) |
| GBS | Glucose | LAZ-2M | 0.60741 (0.13052~1.11, 0.008**) | 0.41324 (-0.07901~0.89, 0.098.) | 0.19417 (0.00384~0.48, 0.048*) | 0.3197 | 31.026% (-0.00284~1.33, 0.056.) |
| GBS | Streptococcus | Octanoylcarnitine | -5.8159 (-9.3772~-2.43, <2e-16***) | -4.0457 (-7.3769~-1.20, 0.012*) | -1.7702 (-3.8082~-0.18, 0.032*) | 0.3044 | 29.32% (0.0355~0.71, 0.032*) |
| GBS | Streptococcaceae | Octanoylcarnitine | -5.8345 (-9.1062~-2.47, <2e-16***) | -3.9751 (-7.1304~-0.89, 0.012*) | -1.8593 (-4.0068~-0.28, 0.018*) | 0.3187 | 31.88% (0.0544~0.75, 0.018*) |
| GBS | Bacteroides | Glucose | 17584.467 (1600.075~33671.36, 0.034*） | 10375.417 (-2682.130~22782.39, 0.140) | 7209.049 (-1163.228~17060.96, 0.092.) | 0.4100 | 39.50% (-0.210~1.46, 0.102) |
| GBS | Bacteroidaceae | Glucose | 17935.778 (2427.028~33182.87, 0.014*) | 10648.468 (-2251.573~23206.82, 0.114) | 7287.310 (-880.811~17278.10, 0.090.) | 0.4063 | 40.7% (-0.092~1.35, 0.092.) |
| GBS | Bacteroides | 10Z-Nonadecenoic acid | 7.0652 (-0.9205~15.90, 0.088.) | 7.7839 (0.0454~16.72, 0.050*) | -0.7187 (-3.0998~1.07, 0.438) | -0.1017 | -6.41% (-1.1403~0.87, 0.498) |
| GBS | Bacteroidaceae | 10Z-Nonadecenoic acid | 0.6486 (0.2344~1.08, <2e-16***) | 0.6772 (0.2367~1.12, <2e-16***) | -0.0285 (-0.1712~0.10, 0.61) | -0.0439 | -3.07% (-0.3657~0.19, 0.61) |
| GBS | Bacteroidales | 10Z-Nonadecenoic acid | 7.3095 (-0.2929~15.20, 0.06.) | 8.1107 (0.0319~15.76, 0.05*) | -0.8012 (-3.7910~1.59, 0.52) | -0.1096 | -8.45% (-1.5901~0.54, 0.55) |
| GBS | Bacteroides | Octanoylcarnitine | -5.8203 (-9.4677~-2.34, <2e-16***) | -5.3615 (-8.8356~-2.01, <2e-16***) | -0.4588 (-1.7116~0.31, 0.27) | 0.0788 | 6.69% (-0.0631~0.29, 0.27) |
| GBS | Bacteroidaceae | Octanoylcarnitine | -5.7662 (-9.2294~-2.05, 0.006**) | -5.3135 (-8.8288~-1.85, 0.008**) | -0.4527 (-1.6448~0.28, 0.286) | 0.0785 | 6.45% (-0.0530~0.35, 0.288) |
| GBS | Bacteroidetes | Octanoylcarnitine | -5.8220 (-9.2110~-2.25, 0.002**) | -5.1241 (-8.6133~-1.45, 0.002**) | -0.6979 (-2.0694~0.30, 0.190) | 0.1199 | 11.10% (-0.0484~0.46, 0.188) |
| GBS | Bacteroidales | Octanoylcarnitine | -5.8704 (-9.3391~-2.63, <2e-16***) | -5.2090 (-8.6968~-1.80, 0.004**) | -0.6614 (-2.1623~0.26, 0.192) | 0.1127 | 10.01% (-0.0426~0.41, 0.192) |
| GBS | Bacteroidia | Octanoylcarnitine | -5.8160 (-9.6237~-2.47, 0.002**) | -5.1364 (-8.6284~-1.79, 0.006**) | -0.6796 (-2.0397~0.24, 0.164) | 0.1169 | 10.73% (-0.0525~0.41, 0.166) |
| GBS | Lactobacillales | Octanoylcarnitine | -5.8160 (-9.6237~-2.47, 0.002**) | -5.1364 (-8.6284~-1.79, 0.006**) | -0.6796 (-2.0397~0.24, 0.164) | 0.1169 | 10.73% (-0.0525~0.41, 0.166) |
| GBS | Lactobacillus | Octanoylcarnitine | -5.8911 (-9.3704~-2.78, 0.002**) | -5.6254 (-9.1353~-2.51, <2e-16***) | -0.2656 (-1.2255~0.45, 0.498) | 0.0451 | 3.28% (-0.0962~0.24, 0.496) |
| GBS | Lactobacillaceae | Octanoylcarnitine | -5.9343 (-9.2745~-2.58, 0.002**) | -5.6392 (-9.1403~-2.11, <2e-16***) | -0.2951 (-1.3033~0.36, 0.432) | 0.0497 | 3.48% (-0.0765~0.25, 0.430) |
| GBS | Flavobacteriaceae | LAZ-2M | 0.6470 (0.2255~1.07, 0.004**) | 0.5773 (0.1353~1.02, 0.014*) | 0.0697 (-0.0708~0.25, 0.298) | 0.1077 | 9.37% (-0.1359~0.52, 0.302) |
| GBS | Flavobacteriales | LAZ-2M | -0.4692 (-0.9051~0.01, 0.052.) | -0.3885 (-0.8449~0.10, 0.124) | -0.0807 (-0.2656~0.07, 0.268) | 0.1720 | 14.23% (-0.3467~1.43, 0.312) |
| GBS | Lactobacillus | LAZ-2M | 0.6499 (0.2058~1.08, 0.004**) | 0.6307 (0.2258~1.06, 0.004**) | 0.0192 (-0.0745~0.13, 0.678) | 0.0295 | 1.85% (-0.1793~0.22, 0.678) |
| GBS | Lactobacillaceae | LAZ-2M | 0.6499 (0.2058~1.08, 0.004**) | 0.6307 (0.2258~1.06, 0.004**) | 0.0192 (-0.0745~0.13, 0.678) | 0.0295 | 1.85% (-0.1793~0.22, 0.678) |
| GBS | Lactobacillales | LAZ-2M | 0.6452 (0.1974~1.08, 0.002**) | 0.6133 (0.1646~1.03, 0.004**) | 0.0319 (-0.0502~0.17, 0.538) | 0.0494 | 3.64% (-0.1085~0.27, 0.536) |
| GBS | Streptococcaceae | LAZ-2M | 0.6572 (0.2408~1.13, 0.002**) | 0.6850 (0.2628~1.14, <2e-16***) | -0.0278 (-0.1654~0.10, 0.638) | -0.0423 | -3.20% (-0.3572~0.15, 0.640) |
| GBS | Streptococcus | LAZ-2M | 0.6486 (0.2344~1.08, <2e-16***) | 0.6772 (0.2367~1.12, <2e-16***) | -0.0285 (-0.1712~0.10, 0.61) | -0.0439 | -3.07% (-0.3657~0.19, 0.61) |
| GBS | 10-Trans-Heptadecenoic acid | LAZ-2M | 0.613036 (0.155684~1.09, 0.01**) | 0.613902 (0.131532~1.09, 0.01**) | -0.000867 (-0.136236~0.14, 0.97) | -0.0014 | -0.1708% (-0.383840~0.28, 0.97) |
| GBS | Octanoylcarnitine | LAZ-2M | 0.6189 (0.1562~1.10, 0.010**) | 0.5185 (0.0476~0.99, 0.032*) | 0.1004 (-0.0641~0.36, 0.250) | 0.1622 | 14.38% (-0.1404~0.82, 0.248) |
| GBS | 10Z-Nonadecenoic acid | LAZ-2M | 0.6297 (0.0955~1.12, 0.018*) | 0.5700 (0.0854~1.04, 0.026*) | 0.0597 (-0.1061~0.29, 0.510) | 0.0948 | 6.97% (-0.2985~0.54, 0.520) |
| GBS | Bacteroidetes | 10Z-Nonadecenoic acid | 7.2982 (-0.4693~15.82, 0.068.) | 8.0375 (-0.2147~16.31, 0.058.) | -0.7393 (-3.7584~1.38, 0.540) | -0.1013 | -7.47% (-1.6835~0.97, 0.580) |
| GBS | Bacteroidia | 10Z-Nonadecenoic acid | 7.3599 (-0.9358~15.67, 0.092.) | 8.0852 (-0.4983~16.18, 0.066.) | -0.7253 (-3.4110~1.62, 0.516) | -0.0985 | -6.62% (-1.2103~0.97, 0.564) |
| GBS | Flavobacteriales | 10-Trans-Heptadecenoic acid | 103.8772 (-6.0261~221.22, 0.068.) | 96.2300 (-17.4927~210.75, 0.084.) | 7.6472 (-27.9179~48.92, 0.688) | 0.0736 | 6.06% (-0.6086~0.94, 0.692) |
| GBS | Flavobacteriaceae | 10-Trans-Heptadecenoic acid | 105.0184 (-2.5336~212.07, 0.07.) | 95.9132 (-18.2391~207.74, 0.11) | 9.1052 (-24.4958~49.39, 0.64) | 0.0867 | 6.48% (-0.9236~1.18, 0.67) |
| GBS | Streptococcus | 10-Trans-Heptadecenoic acid | 105.836 (-2.701~222.03, 0.062.) | 89.585 (-24.270~205.23, 0.128) | 16.251 (-12.843~59.04, 0.280) | 0.1535 | 12.7% (-0.381~1.27, 0.322) |
| GBS | Streptococcaceae | 10-Trans-Heptadecenoic acid | 105.813 (-0.590~206.92, 0.052.) | 89.230 (-15.658~197.35, 0.098.) | 16.583 (-9.923~58.98, 0.274) | 0.1567 | 13.9% (-0.381~0.95, 0.306) |
| GBS | Lactobacillales | 10-Trans-Heptadecenoic acid | 105.7260 (-6.0871~210.66, 0.068.) | 101.6165 (-9.3250~211.48, 0.080.) | 4.1095 (-19.2679~35.28, 0.754) | 0.0389 | 20.5% (-0.5466~0.57, 0.770) |
| GBS | Lactobacillus | 10-Trans-Heptadecenoic acid | 102.8184 (-12.4749~216.5, 0.08.) | 91.1554 (-20.6916~197.5, 0.10) | 11.6630 (-8.8097~48.4, 0.39) | 0.1134 | 7.72% (-0.2206~0.7, 0.41) |
| GBS | Lactobacillaceae | 10-Trans-Heptadecenoic acid | 103.9165 (-6.2192~206.12, 0.062.) | 92.3622 (-15.4701~195.76, 0.090.) | 11.5543 (-10.7748~45.84, 0.350) | 0.1112 | 8.25% (-0.2072~0.82, 0.368) |

*Note*. X, independent variable; M, mediator; Y, dependent variable; ADE, average direct effects; ACME, average causal mediation effects (indirect effect); CI, Confidence Interval. LAZ-2M, length-for-age *z*-score of infants at 2 months of age.

**Table S5.** Markers and discrimination for distinguishing LAZ<-1 in infants aged 1-8 months selected by LASSO

| Data sources | Variables that were LASSO selected and passed the test of proportional hazard assumption in COX models | *N* | C-index |
| --- | --- | --- | --- |
| baseline data | Hospital, Maternal height and weight before delivery | 3 | 0.713 |
| birth data | IAP doses, Duration from birth to discharge LAZ, WAZ, BMIAZ at birth | 5 | 0.812 |
| baseline+birth data | Height of mother, LAZ and WAZ at birth | 3 | 0.817 |
| VM | No qualified variables | / | / |
| VM+baseline +birth data | Height of mother, LAZ and WAZ at birth，abundance of *Actinotignum timonense* | 4 | 0.868 |
| FGMF1 | abundance of *Lactobacillales, Tissierellaceae, Blastococcus,* and *Actinobacillus* | 4 | 0.598 |
| FMGF1+ baseline +birth data | LAZ and WAZ at birth，abundance of *Blastococcus* | 3 | 0.869 |
| Q300F1 | Oxalic acid | 1 | 0.676 |
| Q300F1+ baseline +birth data | LAZ at birth，3-hydroxybutyrate，Oxalic acid | 3 | 0.883 |
| FGMF1+Q300F1 | Oxalic acid | 1 | 0.676 |
| FGMF1+Q300F1+b baseline +birth data | LAZ at birth, 3-hydroxybutyrate, Oxalic acid | 3 | 0.883 |

*Note*. VM, vaginal microbiota in the third trimester; FGMF1, gut microbiota of infants aged 2-3 days; Q300F1, metabolites of infants aged 2-3 days. IAP dose, dose of maternal intrapartum antibiotic prophylaxis; LAZ, length-for-age *z*-score; WAZ, weight-for-age z-score; BMI *z*-score, body mass index-for-age *z*-score. *N*, number of variables used in the model.

**Table S6.** Markers and discrimination for distinguishing LAZ<-1 in infants aged 0-8 months selected by LASSO

| Data sources | Variables that were LASSO selected and passed the test of proportional hazard assumption in COX models | *N* | C-index |
| --- | --- | --- | --- |
| Baseline | GBS test, Height and weight of mother at third trismester | 3 | 0.658 |
| VM | abundance of *Lactobacillus coleohominis*, *Anaerococcus*, *uncultured microorganism* | 3 | 0.598 |
| VM+baseline | Hospital, Height of mother, abundance of *Lactobacillus coleohominis*, *Aerococcus and uncultured microorganism* | 5 | 0.712 |

*Note*. VM, vaginal microbiota in the third trimester; C-index, concordance-index. *N*, number of variables used in the model.
