# Supplementary figures and images for: Maternal group B Streptococcus decreases infant length and alters the early-life microbiome: a prospective cohort study
Source: Ann Med. 2024 Dec 18;57(1):2442070. doi: 10.1080/07853890.2024.2442070 (PMC11656753; doi:10.1080/07853890.2024.2442070)

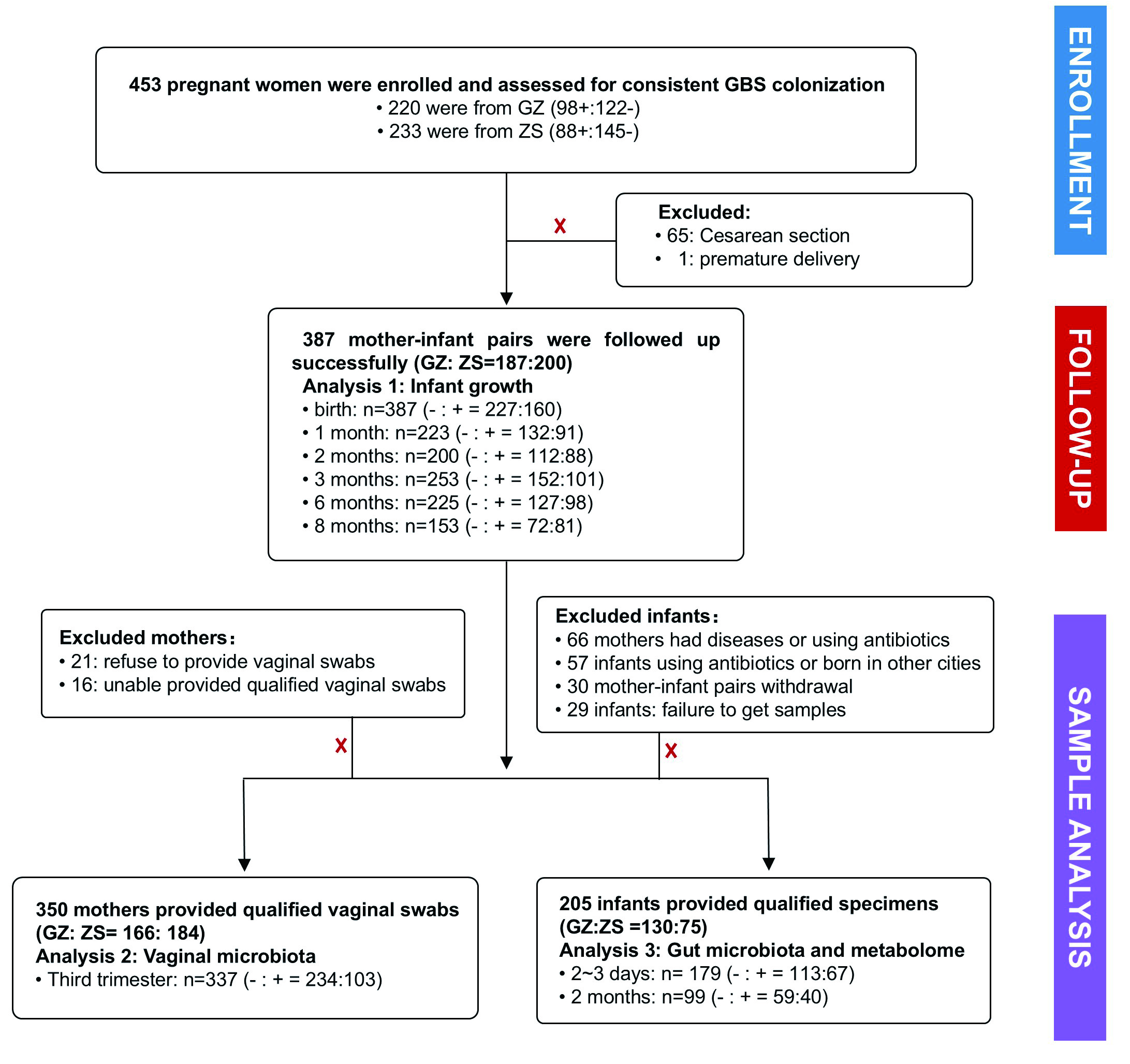

Supplement: Supplemental Material [file IANN_A_2442070_SM4854.zip › suppl_data/Figure_S1.jpg]

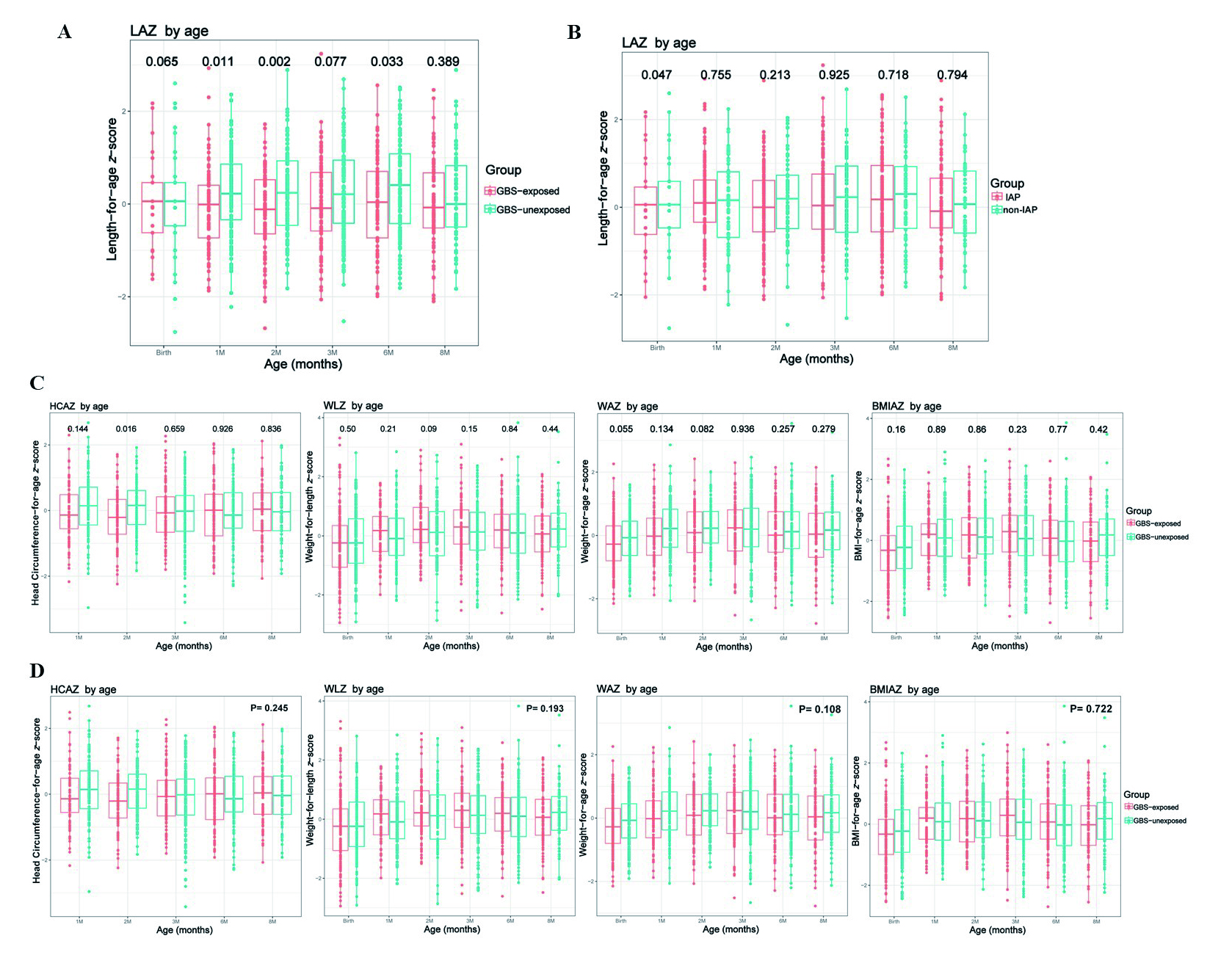

Supplement: Supplemental Material [file IANN_A_2442070_SM4854.zip › suppl_data/Figure_S2.jpg]

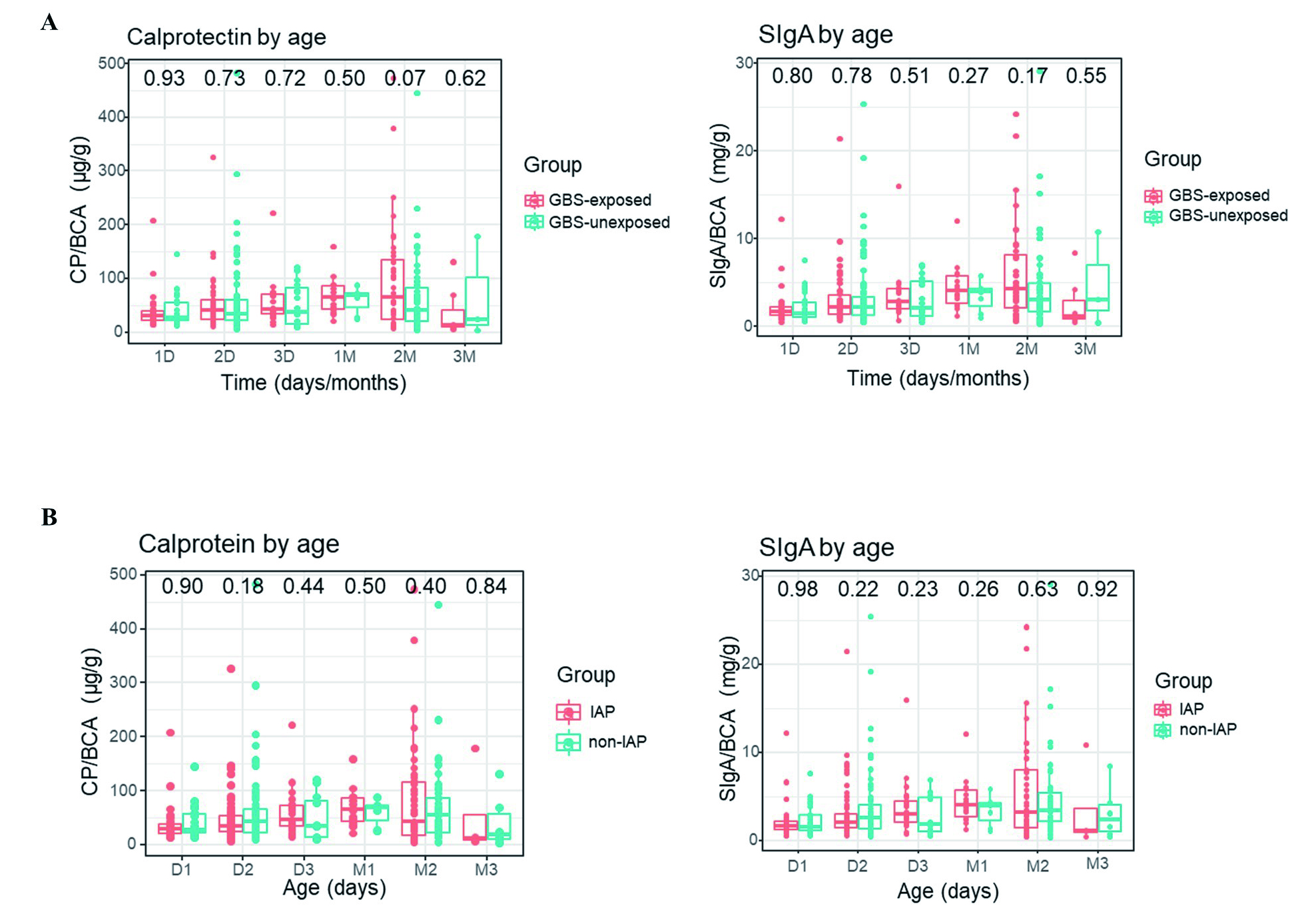

Supplement: Supplemental Material [file IANN_A_2442070_SM4854.zip › suppl_data/Figure_S3.jpg]

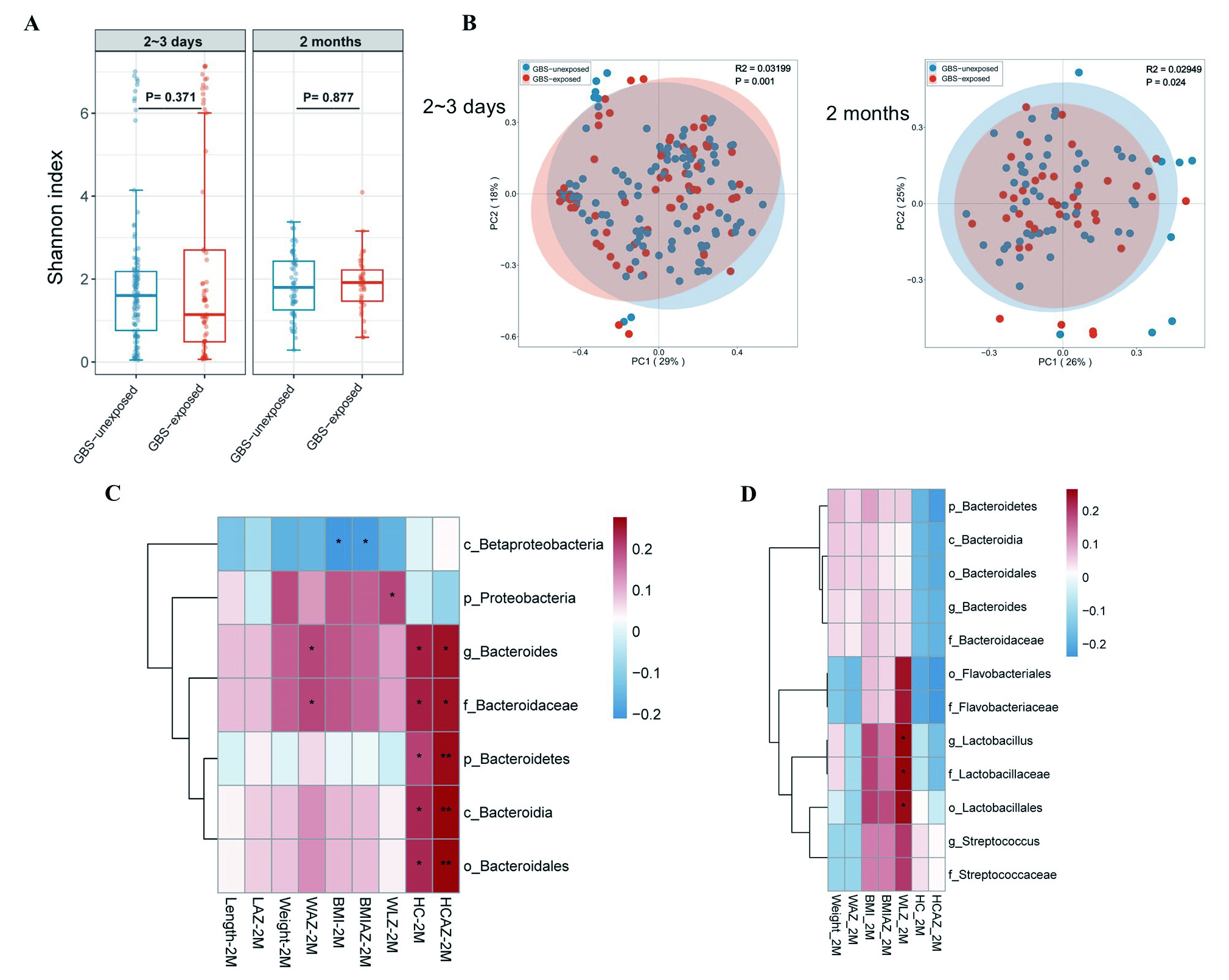

Supplement: Supplemental Material [file IANN_A_2442070_SM4854.zip › suppl_data/Figure_S4.jpg]

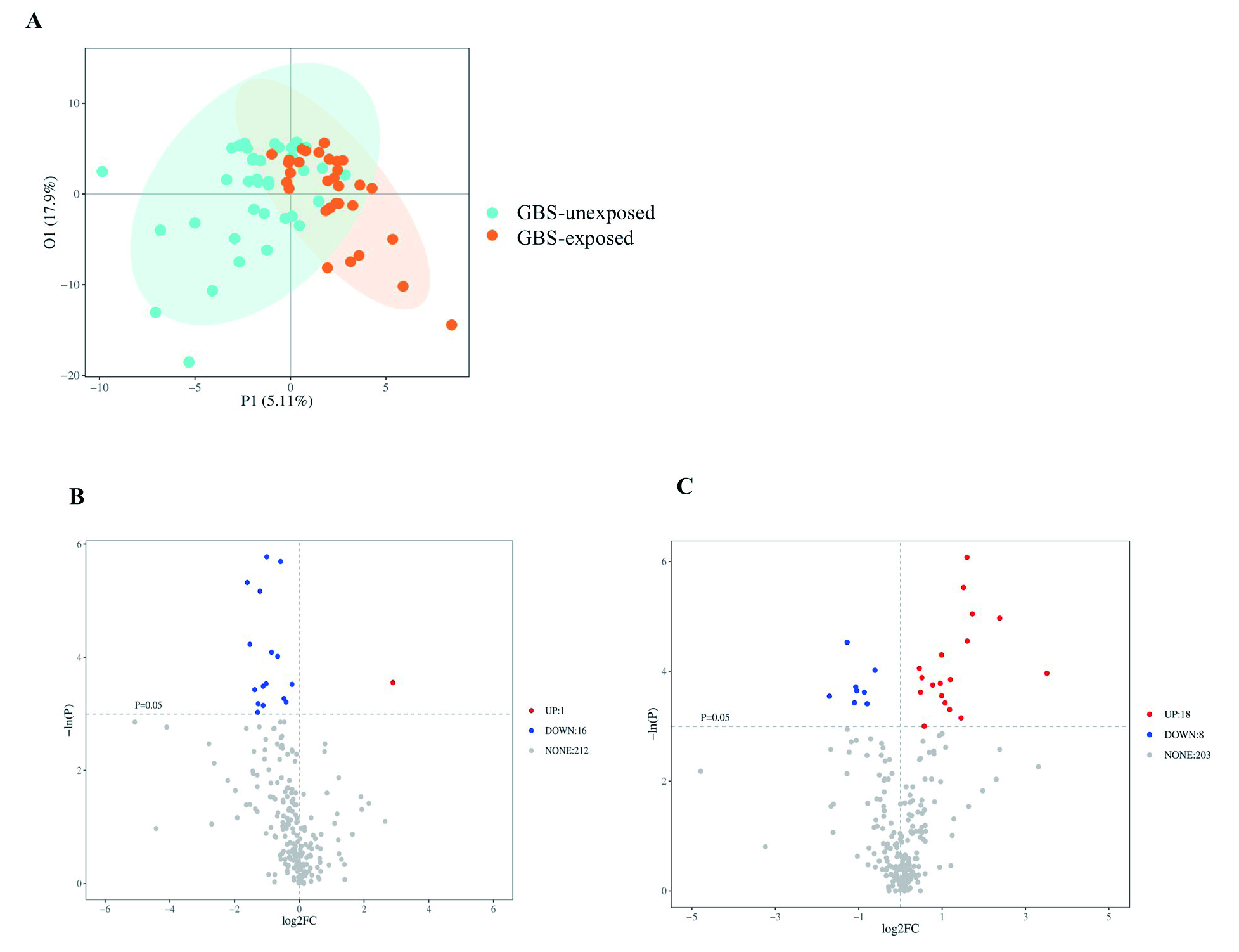

Supplement: Supplemental Material [file IANN_A_2442070_SM4854.zip › suppl_data/Figure_S5.jpg]

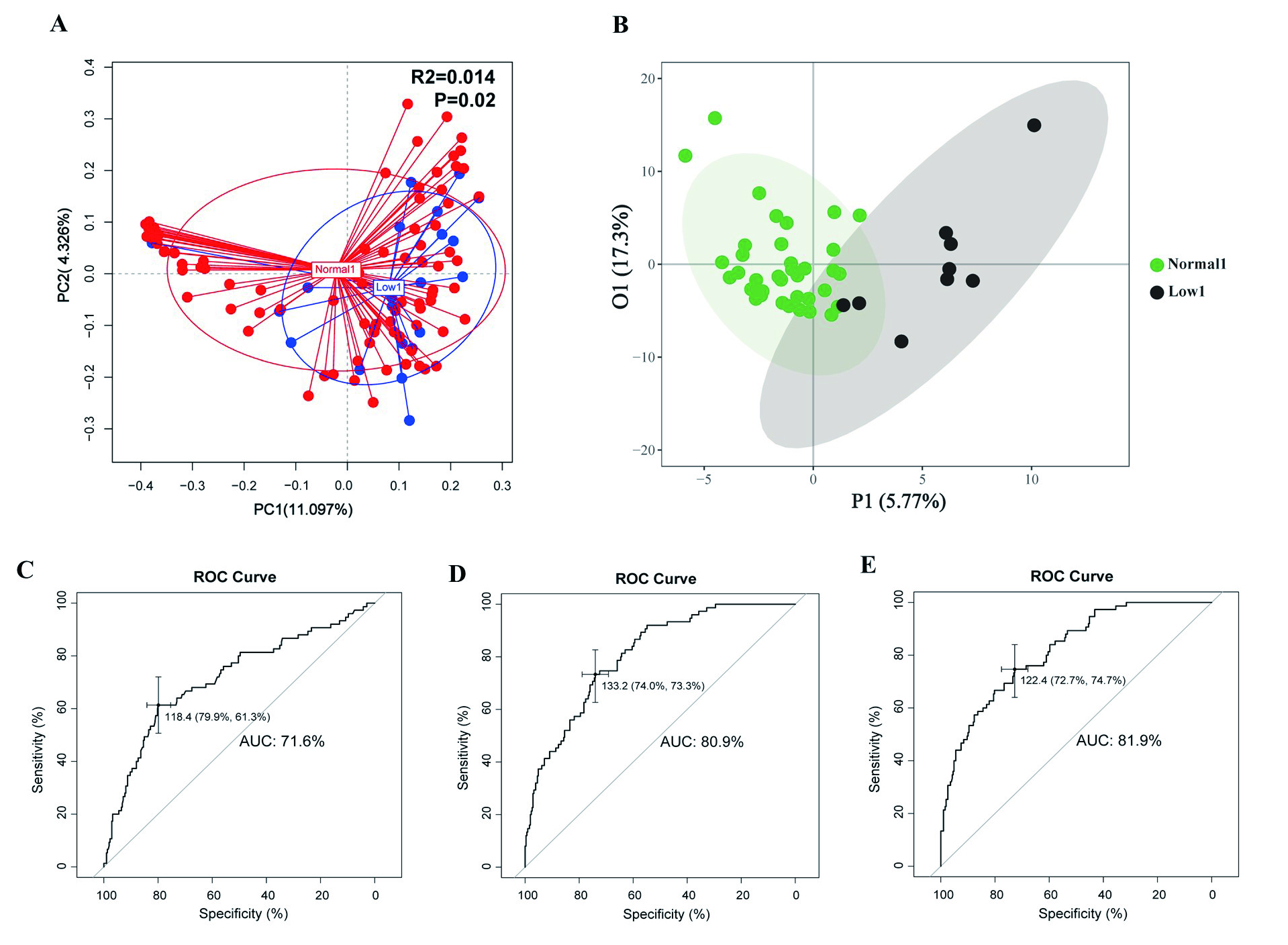

Supplement: Supplemental Material [file IANN_A_2442070_SM4854.zip › suppl_data/Figure_S6.jpg]

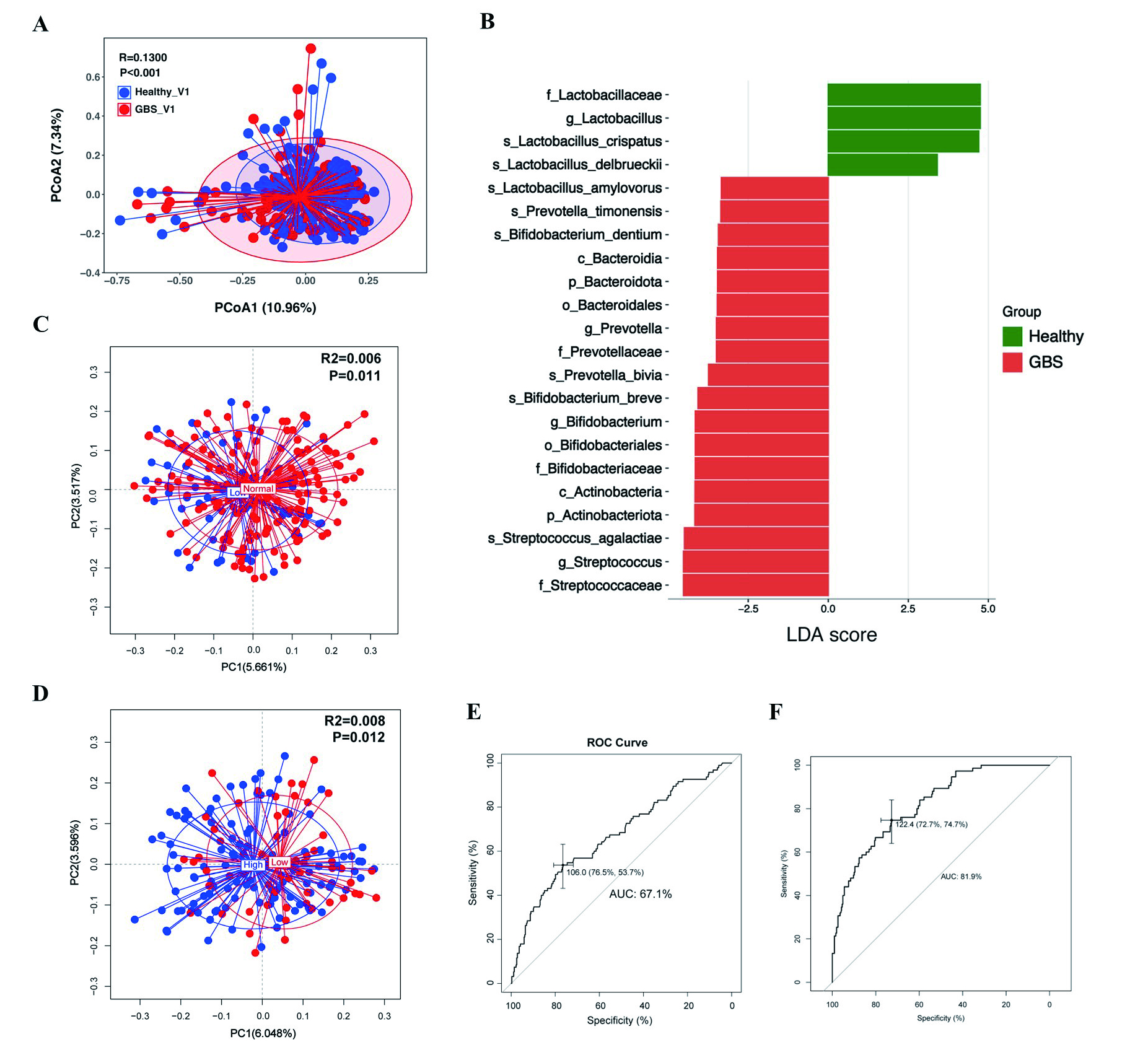

Supplement: Supplemental Material [file IANN_A_2442070_SM4854.zip › suppl_data/Figure_S7.jpg]
